# Improved performance of a rainbow trout selected strain is associated with protein digestion rates and synchronization of amino acid absorption

**DOI:** 10.1038/s41598-020-61360-0

**Published:** 2020-03-13

**Authors:** Andreas Brezas, Ronald W. Hardy

**Affiliations:** Hagerman Fish Culture Experiment Station, University of Idaho, 3059F National Fish Hatchery Road, Hagerman, ID 83332 USA

**Keywords:** Biological techniques, Physiology

## Abstract

Replacement of fishmeal in feeds is critical for sustainable aquaculture growth. However, replacement with plant protein concentrates reduces fish performance. A rainbow trout strain selected for high performance on a plant protein diet was compared to a non-selected strain to identify physiological mechanisms associated with improved performance. Nutrient digestibility in fishmeal and plant protein diets was assessed and no strain differences were found. Levels of amino acids in the hepatic portal vein and caudal vein were measured at intervals after a single force-feeding of fishmeal, four plant protein concentrates, and a mixture of the concentrates with or without supplementation of three limiting amino acids. Each ingredient affected plasma amino acid levels in a singular manner when fed individually but without predictable additive effects when fed as a mixture. Amino acid supplementation altered uptake and plasma concentrations of all the essential amino acids. The selected trout strain fed the plant protein mixture with amino acids showed a synchronous and homogenous pattern for essential amino acids over time in the hepatic portal vein in contrast to that of the non-selected strain. The results demonstrate that selection favorably altered temporal dynamics of plant protein digestion.

## Introduction

Replacement of fishmeal as the major protein source in feeds is critical for continued growth of the aquaculture industry as well as advancement of sustainable aquaculture^1–3^. Plant protein concentrates produced from grains, oilseeds and pulses are the leading alternative protein sources to replace fishmeal in fish feeds. However, numerous studies have shown suboptimal fish growth performance and reduced protein retention efficiency when carnivorous fish species are fed low-fishmeal, high-plant protein feeds even when all known dietary essential nutrients, including amino acids, are present above required levels^4–8^. There are several factors blamed for reduced growth of carnivorous fish fed plant protein-based diets, including reduced feed intake, antinutrients in plant products, differences in levels of biologically significant components, such as anabolic steroids or phytoestrogens in plant proteins compared to fishmeal, unidentified nutrient deficiencies and an imbalance of essential amino acids^9–11^ In the case of the later, plant proteins generally have less lysine, methionine and threonine compared to fishmeal and are often deficient compared to dietary requirements of fish^3^. To correct deficiencies in plant protein-based diets, amino acid supplements are added^3^. However, evidence suggests that this approach may cause an imbalance of amino acids in blood plasma associated with rapid uptake of supplemented amino acids and delayed digestion and absorption of amino acids of plant origin compared to fishmeal^12–14^. Protein synthesis in cells requires that all essential amino acids are available at the moment proteins are synthesized; if one essential amino acid is not present in sufficient amounts, the remaining amino acids are alternatively metabolized for energy^3^. This may result in lower protein retention efficiency and increased protein turnover, a common observation when fish are fed plant-based feeds^5–7,13^.

A rainbow trout strain (ARS/KO) has been developed using selective breeding over 12 years (six generations) based on growth performance when fed an all-plant protein feed at the University of Idaho in collaboration with the US Department of Agriculture’s Agricultural Research Service. The selected strain grows rapidly and efficiently when fed all plant-protein feeds containing 45% soy products, unlike non-selected trout that exhibit 10–15% lower growth and feed efficiencies than selected trout^15^. The selected strain can be considered as a model to explore and identify physiological parameters associated with improved plant protein utilization in carnivorous fish.

The goal of the present study was to identify digestive mechanisms associated with improved performance of the selected strain when fed an all-plant protein, soy-based diet. To explore digestive mechanisms, we performed two series of experiments. In the first experiment we investigated if selection improved nutrient digestibility by comparing the selected strain with a non-selected fast-growing strain of rainbow trout when fed a fishmeal-based diet and the selection diet (all-plant protein soy-based). In the second experiment, both rainbow trout strains were fed five practical ingredients (fishmeal and four plant protein concentrates) individually or as mixture of the plant protein concentrates with or without amino acid supplementation. Plasma amino acid patterns were measured over time after a single feeding to investigate if plasma amino acid temporal patterns at the absorption site, the hepatic portal vein (HPV), and from the systemic blood, the caudal vein (CV), could reveal differences between the two trout strains associated with growth performance and be used to assess alternate ingredients. Further, we investigated if results obtained with single feed ingredients could predict nutritional value when the alternate ingredients were combined and if supplementing a plant protein mixture with limiting amino acids influenced absorption and apparent utilization of other amino acids. Finally, we tested the hypothesis that the improved protein utilization and growth demonstrated by the selected strain when fed an all-plant protein soy-based diet was associated with synchronized amino acid uptake.

## Materials and Methods

### Experiment 1: Digestibility trial

#### Experimental diets

*In vivo* digestibility values for fishmeal (FMD) and plant meal-based (PMD) diets in two different strains were determined by feeding selected and non-selected rainbow trout groups the experimental diets containing 0.1% yttrium oxide as an indigestible inert marker (Table 1). Diets were cold-pelleted at the Hagerman Fish Culture Experiment Station (HFCES) using a California pellet mill fitted with a 4.0 mm die. The pellets were forced-air dried at 37 °C for 48 h to less than 10% moisture. Samples of each diet were collected for analysis.

**Table 1:** Composition of the experimental diets (g/100 g).

Ingredient	Plant meal Diet	Fishmeal Diet
Soy protein concentrate	25.63	15.00
Soybean meal	19.55	
Corn protein concentrate	17.54	10.00
Wheat gluten meal	4.07	7.00
Wheat starch	8.81	18.00
Fish meal		33.00
Fish oil	15.70	14.00
L-Lysine	1.40	
DL-Methionine	0.38	
Threonine	0.20	
Taurine	0.50	
Dicalcium phosphate	3.33	1.20
Potassium chloride	0.56	
Sodium chloride	0.28	
Magnesium oxide	0.05	
Stay-C (35% ascorbate)	0.20	0.20
Choline chloride	0.60	0.60
Yttrium oxide	0.10	0.10
Trace mineral premix^a^	0.10	0.10
Vitamin premix 702^b^	1.00	0.80

^a^Supplied the following per kg diet: copper, 3 mg as copper sulfate pentahydrate; manganese, 10 mg as manganese sulfate monohydrate; iodine, 5 mg as potassium iodide; sodium selenate 0.960 mg; and zinc, 37 mg as zinc sulfate, heptahydrate.

^b^Supplied the following per kg diet: vitamin A palmitate, 9650 IU; cholecalciferol, 6600 IU; DL-tocopheryl acetate, 132 IU; menadione sodium bisulfate 1.1 mg; thiamin mononitrate 9.1 mg; riboflavin 9.6 mg; pyridoxine HCl 13.7 mg; DL-calcium pantothenate, 46.5 mg; cyanocobalamine 0.03 mg, nicotinic acid, 21.8 mg; D-biotin, 0.34 mg; folic acid 2.5; and inositol, 600 mg.

#### Fish and feeding

Rainbow trout from broodstock (House Creek and ARS/KO strains) maintained at HFCES were used in the digestibility study. Eight groups of 35 fish (average body weight 228 g) were stocked into eight 450 l tanks supplied with 25 l min^−1^ of constant temperature spring water (15 °C). Each diet was randomly assigned to two replicate tanks of fish per strain. Fish were fed their respective diets twice daily to apparent satiation for eight days. Photoperiod was maintained at a constant 14 h light: 10 h dark with a timer controlling fluorescent lights. Fish were acclimated to the experimental diets for four days, and then on days five and nine, all the fish from each tank were lightly anaesthetized using tricaine methanosulfonate (MS-222, 100 mg/L, buffered to pH 7.0), removed from water, and feces were gently expelled using light pressure on the abdomen near the vent, a process called stripping. Care was taken to avoid contamination of feces with urine from the fish. Fecal samples were collected in aluminum pans, pooled by tank and were frozen between strippings. Two strippings generated 71–112 g wet fecal samples which yielded 11–14 g dry feces per tank, sufficient material for subsequent chemical analysis. All protocols used in the digestibility trial were approved in advance by the University of Idaho’s Institutional Animal Care and Use Committee in accordance with relevant guidelines and regulations.

#### Chemical analysis

Experimental feeds and fecal samples from both strains were analyzed for proximate composition, mineral and amino acid levels and energy content^16^. Samples were dried in a convection oven at 105 °C for 12 h to determine moisture level according to AOAC^17^. Dried samples were finely ground by mortar and pestle and analyzed for crude protein (total nitrogen x 6.25) using combustion method with a nitrogen determinator (TruSpec N, LECO Corporation, St. Joseph, MI). Crude lipid was analyzed by subjecting samples to acid hydrolysis using an ANKOM HCL hydrolysis system (ANKOM Technology, Macedon, NY) and extracting them with petroleum ether using an ANKOM XT15 extractor. Ash was analyzed by incineration at 550 °C in a muffle furnace for 5 h. Energy content of samples was determined using an isoperibol bomb calorimeter (Parr 6300, Parr Instrument Company Inc., Moline, IL). Yttrium was measured in feeds and feces samples from the digestibility trial by inductively-coupled plasma (ICP) analysis at the University of Idaho Analytical Sciences Laboratory, Moscow, ID. Amino acid levels in feeds and fecal samples were hydrolyzed and analyzed with an amino acid analyzer (Hitachi Amino Acid Analyzer L-8800) by the University of Missouri’s Agricultural Experiment Station Chemical Laboratories, Columbia, MO. Chemical analyses were done in duplicate.

Apparent Digestibility Coefficients (ADCs) for dry matter, organic matter, protein, amino acids, lipid and energy, were calculated using the following equation described by Bureau *et al*.^18^.

$${\rm{ADC}}\,{\rm{diets}}=1-[({\rm{F}}/{\rm{D}})\times ({\rm{Di}}/{\rm{Fi}})]$$

where D = % nutrient of diet, F = % nutrient of feces, Di = % digestion indicator of diet, Fi = % digestion indicator of feces.

### Experiment 2

#### Experimental fish and dietary treatments

Two strains of rainbow trout were used, the selected strain (ARS/KO strain) and a non-selected (House Creek strain)^15^. Both strains originated from broodstock held at the HFCES. Three hundred and fifty individuals (175/strain) with an average weight 580 ± 209 g were distributed randomly in 70 tanks (5 individuals/strain/tank). The tank water volume was 144 l and each tank was supplied with 12 l min^−1^ of constant temperature spring water (15 °C) under a controlled photoperiod (14 h light: 10 h dark). The study was conducted over a period of three weeks such that for every day of sampling 10 tanks per test diet were used. Prior to experimental use, the fish were hand fed to apparent satiation with a commercial diet (Skretting, USA), then fasted for two days. All protocols used in the trial were approved in advance by the University of Idaho’s Institutional Animal Care and Use Committee in accordance with relevant guidelines and regulations.

#### Diets

Five practical, high-protein ingredients were used in the feeding experiment, anchovy fish meal (FM); corn protein concentrate (CPC); soybean meal (SBM); soy protein concentrate (SPC) and wheat gluten meal (WGM). A plant-protein soy-based mixture was prepared from the ingredients to duplicate the protein composition of the selection diet used to develop the ARS/KO trout strain. The mixture was either supplemented with crystalline amino acids (lysine, methionine and threonine) in proportions equal to those used in the selection diet (Diet Plus) or without amino acid supplementation (Diet Minus) (Table 2).

**Table 2:** Composition of the experimental plant-protein mixtures (g/100 g).

Ingredient	Diet Minus	Diet Plus
Soy protein concentrate	38.37	37.27
Soybean meal	29.27	28.43
Corn protein concentrate	26.26	25.51
Wheat gluten meal	6.09	5.92
L-Lysine		2.04
DL-Methionine		0.55
Threonine		0.29

#### Force feeding

The force-feeding procedure followed that of Ambardekar *et al*.^13^ with minor modifications. After a period of 48 h fasting and prior to gavage, trout were lightly anesthetized (40 mg/l MS-222, buffered to pH 7.0) and weighed. Each of the test ingredients was mixed with two parts water to create a slurry and delivered to the fish by stomach intubation at 0.5% of live body weight (ratio of dry ingredient or blend to wet body weight). Each anesthetized fish was forced fed the diet slurry with a 60 ml syringe attached to a piece of Tygon tubing long enough to reach the stomach of the fish. After feeding, fish were placed in a vigorously aerated freshwater rinse tank for several minutes and then were returned to their holding tank.

#### Blood sampling

Blood samples were only taken from individuals that did not show signs of slurry regurgitation. Blood sampling points were set at 3, 6, 12, 18 and 24 h post force-feeding (for each time point a different tank with fish was used). Approximately 5 min before blood sampling, each fish was anesthetized in 100 mg/l buffered MS – 222 to heavy sedation, i.e., stage 4 when gill operculum movement slowed. The abdomen was opened, and blood was collected (0.2 to 0.3 ml) from the hepatic portal vein (HPV) with a heparinized winged infusion set (butterfly needle; 12-inch tubing, 23 G and 3/4-inch ultra-thin wall needle) connected to a 1 ml syringe. After gently inverting the syringe 3–4 times for proper mixing, the blood was transferred to a 0.6 ml conical Eppendorf tube on ice. Next, blood was collected (1 to 1.5 ml) from the caudal vein (CV) using a 3-ml heparinized syringe with 22 G 1.5-inch needle. After gently inverting the syringe 3–4 times for proper mixing, the blood was transferred to a 2 ml round bottom Eppendorf tube on ice. Blood samples were centrifuged at 2,000 g for 5 min at 4 °C, and the upper plasma layer collected without red blood cells or buffy coat (white blood cells). Plasma proteins were precipitated by adding 13 μl of sulphosalicylic acid into 130 μl of plasma and mixing by gentle vortex for 5 sec. The samples were then incubated at 4 °C for 20 min and then centrifuged at 16,000 g at 4 °C for 15 min. Deproteinized plasma (105 μl) was then mixed with 30 μl of 0.3 M NaOH. Finally, 28 μl of internal standard (2.5 mM norleucine) and 117 μl sodium citrate loading buffer (pH 2.2) were added and mixed by vortex for 5 sec and then transferred to a spinX 0.2 μm filter tube and centrifuged for 2 min at 15,000 g at room temperature. The retained filtered sample was then analyzed using a Biochrom 30 amino acid analyser (Biochrom LTD Cambridge, UK) according to the manufacturer’s protocol.

### Statistical analysis

#### Experiment 1

Apparent digestibility coefficient values were analyzed for normality (Kolmogorov–Smirnov test) and homoscedasticity (Levene’s test). The interaction of strain and diet effects on dry matter, crude protein, lipid, organic matter, energy and amino acid digestibility were analyzed by two-way ANOVA at a 5% level of significance (α ≤ 0.05). *Post-hoc* tests (Tukey’s HSD test) were performed to identify treatments that differed significantly. Statistical analysis was conducted using Statistica (StatSoft, Tulsa OK, USA).

#### Experiment 2

The five practical ingredients were tested for significant interaction effects of strain and time on plasma amino acid levels. Regarding the plant-protein mixtures with and without amino acid supplementation, we tested if there were significant interaction effects of strain, diet and time on plasma amino acids. Plasma amino acid concentration values were analyzed for normality (Kolmogorov–Smirnov test) and homoscedasticity (Levene’s test). When the assumptions for normality and homoscedasticity were met, multifactorial analysis of variance (ANOVA) was performed using Statistica (StatSoft, Tulsa OK, USA). In the case when data were violating the assumptions, a permutational multivariate analysis of variance (PERMANOVA) was performed using Primer 7 (Primer-E Ltd, Plymouth, UK). *Post-hoc* tests (Student-Newman-Keuls test) were performed to identify treatments that significantly differed. Plasma amino acid concentrations were expressed as the mean of three replicate measurements.

## Results

### Apparent digestibility coefficients (ADCs)

No significant interaction effect between diet and strain for apparent digestibility was detected (Table 3). A statistically significant diet effect was detected for dry matter and crude protein with higher levels reflected in the groups fed the plant-protein based diet. The same pattern was also observed with all individual amino acids (IAA and DAA) and the sums (sum of IAAs and TAAs). The only significant strain effect was proline digestibility (p < 0.05).

**Table 3:** Apparent digestibility coefficients.

	Plant Based Diet		Fishmeal Based Diet		DIET x STRAIN	DIET	STRAIN
	SEL	NON SEL	SEL	NON SEL	P-value	P-value	P-value
Dry Matter	76.4 ± 0.2	77.6 ± 0.3	73.2 ± 1.2	74.2 ± 1.6	ns	P < 0.05	ns
Crude Protein	93.6 ± 0.1	93.5 ± 0.4	85.3 ± 0.7	86.5 ± 0.7	ns	P < 0.001	ns
Lipid	98.0 ± 0.2	97.3 ± 0.1	98.0 ± 0.5	96.1 ± 0.8	ns	ns	ns
Organic Matter	80.2 ± 0.2	81.3 ± 0.4	79.6 ± 1.1	80.5 ± 1.4	ns	ns	ns
Energy	84.1 ± 0.1	84.9 ± 0.3	83.0 ± 1.0	82.9 ± 1.5	ns	ns	ns
Alanine	96.2 ± 0.1	95.6 ± 0.4	90.6 ± 0.5	90.6 ± 0.7	ns	P < 0.001	ns
Arginine	98.4 ± 0.1	98.3 ± 0.2	92.8 ± 0.5	93.2 ± 0.6	ns	P < 0.001	ns
ASX^a^	93.3 ± 0.3	93.0 ± 0.3	86.0 ± 0.8	87.6 ± 0.7	ns	P < 0.001	ns
Cysteine	91.2 ± 0.7	91.8 ± 0.1	81.2 ± 1.0	84.4 ± 1.3	ns	P < 0.001	ns
GLX^b^	97.3 ± 0.1	96.6 ± 0.3	92.3 ± 0.4	92.4 ± 0.6	ns	P < 0.001	ns
Glycine	92.1 ± 0.2	91.4 ± 0.4	82.2 ± 0.7	82.1 ± 1.0	ns	P < 0.001	ns
Histidine	96.5 ± 0.2	96.2 ± 0.2	93.2 ± 0.4	93.6 ± 0.5	ns	P < 0.01	ns
Isoleucine	95.7 ± 0.1	95.1 ± 0.3	91.1 ± 0.5	91.3 ± 0.7	ns	P < 0.001	ns
Leucine	96.9 ± 0.1	96.3 ± 0.3	93.7 ± 0.4	93.9 ± 0.5	ns	P < 0.01	ns
Lysine	97.3 ± 0.2	97.4 ± 0.1	92.4 ± 0.3	93.6 ± 0.5	ns	P < 0.001	ns
Methionine	97.4 ± 0.2	97.4 ± 0.1	91.1 ± 0.3	92.2 ± 0.5	ns	P < 0.001	ns
Phenylalanine	96.8 ± 0.0	96.7 ± 0.1	93.2 ± 0.4	93.2 ± 0.6	ns	P < 0.001	ns
Proline	95.3 ± 0.2	93.0 ± 0.4	87.8 ± 0.2	86.8 ± 0.7	ns	P < 0.001	P < 0.05
Serine	95.7 ± 0.2	95.0 ± 0.6	89.1 ± 0.6	89.5 ± 0.8	ns	P < 0.001	ns
Threonine	92.9 ± 0.3	92.0 ± 0.3	88.1 ± 0.4	88.8 ± 0.8	ns	P < 0.01	ns
Tryptophan	97.0 ± 0.0	97.1 ± 0.0	95.3 ± 0.8	95.4 ± 0.3	ns	P < 0.05	ns
Tyrosine	96.8 ± 0.1	96.4 ± 0.1	92.2 ± 0.6	92.2 ± 0.6	ns	P < 0.001	ns
Valine	94.2 ± 0.1	93.3 ± 0.5	90.4 ± 0.5	90.8 ± 0.7	ns	P < 0.01	ns
Sum AA	95.7 ± 0.2	95.2 ± 0.3	89.7 ± 0.5	90.1 ± 0.7	ns	P < 0.001	ns
Sum EAA (10)	96.5 ± 0.1	96.1 ± 0.2	92.1 ± 0.4	92.6 ± 0.6	ns	P < 0.001	ns

^a^ASX = aspartic acid + asparagine.

^b^GLX = glutamic acid + glutamine.

#### Plasma amino acids

**Figure 1:**
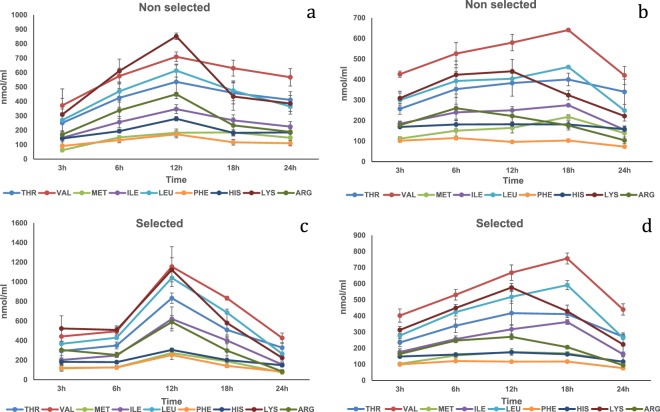
Free essential amino acid (except tryptophan) mean concentrations n = 3 ± SEM in blood plasma (nmol/mL) collected from the hepatic portal vein (**a**,**c**) and caudal vein (**b**,**d**) of two strains of rainbow trout during a 24 h period after force feeding of fishmeal.

Fishmeal. In the HPV, there were significant (P < 0.05) strain and time interactions on Met, Val, Ile and Leu plasma concentrations with both reaching peak levels for the selected strain at 12 h post-prandially (Fig. 1a,c; Table S1). A time effect was significant (P < 0.01) on Thr, Phe, His, Lys and Arg; all peaked also at 12 h post-prandially. In the CV, no interaction was found. Significant differences (P < 0.05) were detected regarding time (all the amino acids) and strain (only His) main effects ((Fig. 1b,d); Table S2). Thr, Met, Val Ile and Leu peaked at 18 h post-prandially while His, Lys and Arg peaked 12 h and Phe at 6 h. However, Val, Ile, Leu, Met, Thr and His recorded significant higher concentrations between 12 and 18 h, while Lys and Arg reached peak levels between 6 and 12 h. Finally, His was significantly higher for the non-selected strain.

**Figure 2:**
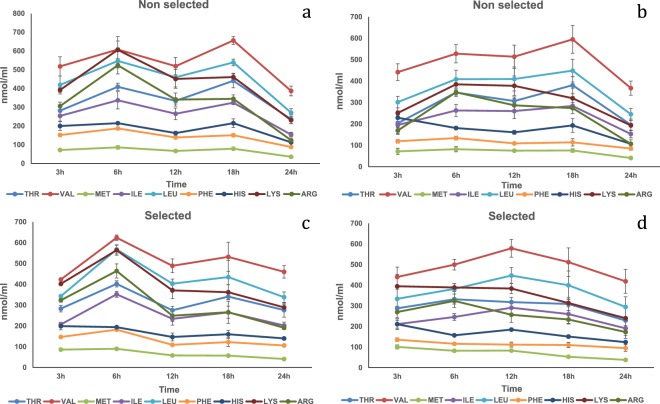
Free essential amino acid (except tryptophan) mean concentrations n = 3 ± SEM in blood plasma (nmol/mL) collected from the hepatic portal vein (**a**,**c**) and caudal vein (**b**,**d**) of two strains of rainbow trout during a 24 h period after force feeding of soy protein concentrate.

Soy protein concentrate. In the HPV, fish fed SPC showed a significant (P < 0.01) peak at 6 h for Thr, Val, Ile, Leu, Met, Phe Lys and Arg (Fig. 2a,c; Table S3). At 18 h a second peak was reached (P < 0.01) for Thr, Val, Ile and Leu. In the CV an interaction was observed regarding Arg (P < 0.05) at 12 h the selected showed significantly lower concentration (Table S4). The time main effect was significant (P < 0.05) for all the amino acids except His. A plateau was observed for all amino acids between 3 and 18 h (except Thr which reached a plateau between 6–18 h) and by 24 h concentrations were below initial baseline levels (Fig. 2b,d).

**Figure 3:**
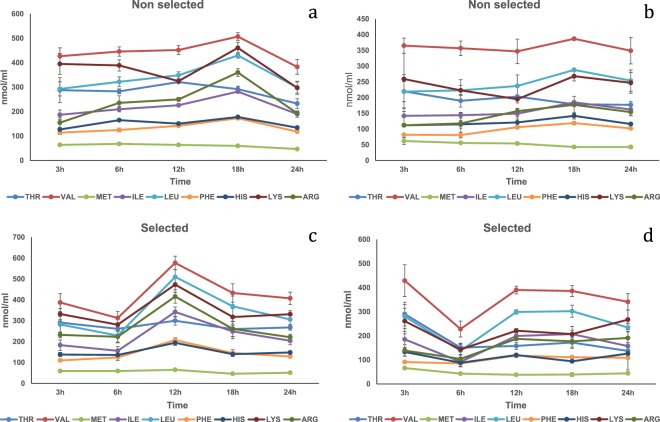
Free essential amino acid (except tryptophan) mean concentrations n = 3 ± SEM in blood plasma (nmol/mL) collected from the hepatic portal vein (**a**,**c**) and caudal vein (**b**,**d**) of two strains of rainbow trout during a 24 h period after force feeding of soybean meal.

Soybean meal. In the HPV, significant interactions (P < 0.01) were detected for all amino acids except Thr and Met (Fig. 3a,c; Table S5). All plasma amino acid concentrations peaked at 12 h in the selected strain and higher compared to the non-selected strain. In addition, the non-selected strain showed a peak at 18 h for Phe, His and Arg higher compared to selected strain. A time effect was observed regarding Met, showing constant levels between 3 and 12 h and dropping later on, reaching its lowest concentration at 24 h post-prandial (P < 0.01). In the CV no interaction was observed (Fig. 3b,d; Table S6). A significant (P < 0.05) time effect was observed. Phe and Arg concentrations at 6 h were significantly lower compared to concentrations between 12 and 24 h, while the concentrations of Ile and Leu at 6 h were significantly lower compared to all other time points. In contrast, Met was the only plasma amino acid that showed the highest concentration only at 3 h compared to the whole monitored period.

**Figure 4:**
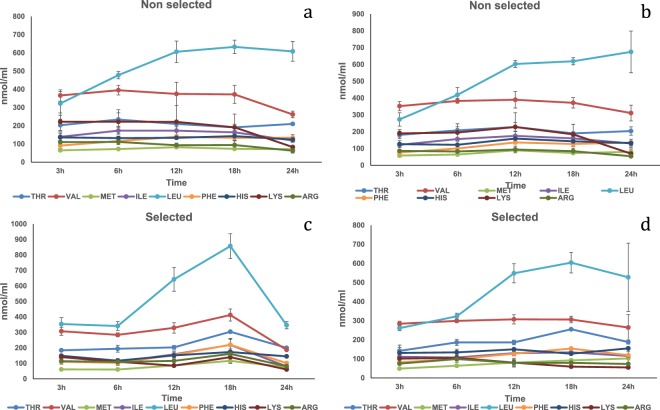
Free essential amino acid (except tryptophan) mean concentrations n = 3 ± SEM in blood plasma (nmol/mL) collected from the hepatic portal vein (**a**,**c**) and caudal vein (**b**,**d**) of two strains of rainbow trout during a 24 h period after force feeding of corn protein concentrate.

Corn protein concentrate. In the HPV, significant (P < 0.05) interactions observed regarding Thr and Leu (Fig. 4a,c; Table S7). The plasma Thr and Leu concentrations in the selected strain peaked at 18 h and were significantly higher compared to the non-selected strain. Time had a significant (P < 0.05) effect on Val, Ile, Leu and Phe. Val and Ile plasma concentrations dropped significantly at 24 h. In contrast, the plasma concentration of Phe at 18 h was significantly higher compared to the 3 h and 6 h time points. A significant main effect (P < 0.05) was observed in Val and Lys concentrations with lower values in the selected strain compared to the non-selected strain. In the CV, no interactions were observed (Fig. 4b,d; Table S8). A significant time effect (P < 0.05) was observed regarding plasma concentrations of Met, Leu and Phe. All the three amino acids showed a significant increase in their concentrations at 12 h. Strain had a significant effect (P < 0.05) on Val, Ile and Lys plasma concentrations being lower in the selected strain.

**Figure 5:**
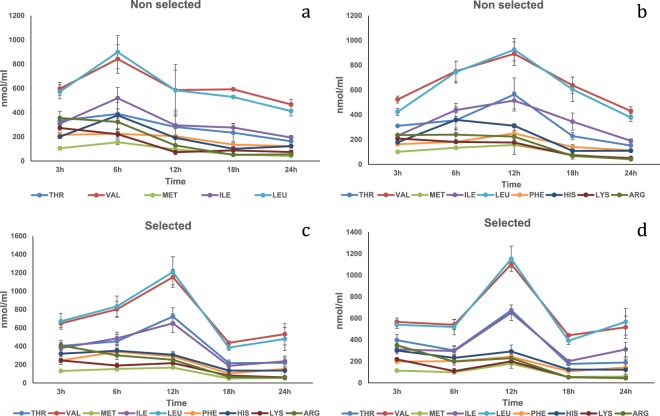
Free essential amino acid (except tryptophan) mean concentrations n = 3 ± SEM in blood plasma (nmol/mL) collected from the hepatic portal vein (**a**,**c**) and caudal vein (**b**,**d**) of two strains of rainbow trout during a 24 h period after force feeding of wheat gluten meal.

Wheat gluten meal. In the HPV, significant interaction effects (P < 0.05) were observed for Thr, Val, Ile, Leu and Lys (Fig. 5a,c; Table S9). All the concentrations of the earlier mentioned amino acids showed higher values at 12 h in the plasma of the selected strain compared to the non-selected strain. Time had a significant effect (P < 0.05) on the concentration of Met, Phe, His and Arg, showing a drop of their levels at 18 h post-prandially. Moreover, a significant strain effect (P < 0.01) was observed for His, with the selected strain having higher concentration levels compared to the non-selected strain. In the CV, significant interaction (P < 0.05) was observed with Val (Fig. 5b,d; Table S10). Val plasma concentration at 12 h post-prandially was higher in the selected strain compared to the non-selected strain. Regarding Thr, Met, Ile and Leu, their plasma concentrations peaked at 12 h post prandially. His, Lys and Arg concentrations dropped significantly at 18 h.

**Figure 6:**
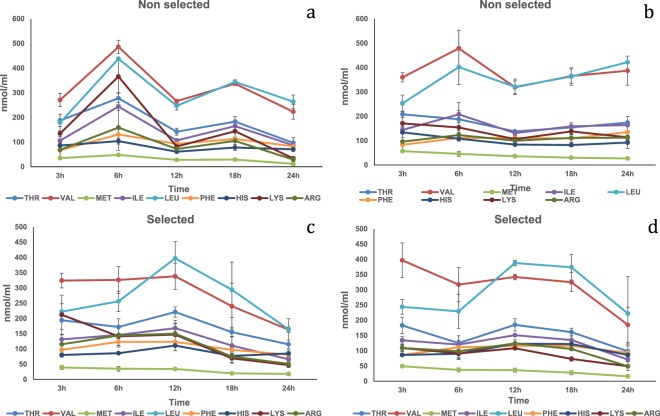
Free essential amino acid (except tryptophan) mean concentrations n = 3 ± SEM in blood plasma (nmol/mL) collected from the hepatic portal vein (**a**,**c**) and caudal vein (**b**,**d**) of two strains of rainbow trout during a 24 h period after force feeding of a plant protein mixture without amino acid supplementation.

**Figure 7:**
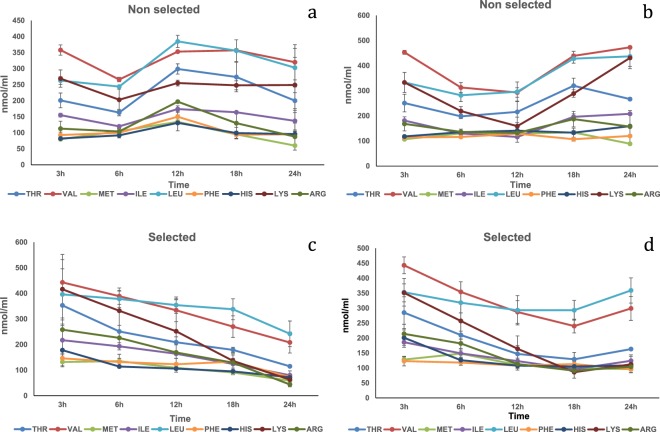
Free essential amino acid (except tryptophan) mean concentrations n = 3 ± SEM in blood plasma (nmol/mL) collected from the hepatic portal vein (**a**,**c**) and caudal vein (**b**,**d**) of two strains of rainbow trout during a 24 h period after force feeding of plant protein mixture with amino acid supplementation.

Plant protein mixtures. In the HPV, a diet by strain by time interaction was significant (P < 0.05) for Thr, Leu, Phe, His and Lys plasma concentrations (Table S11). At 3 h the selected strain fed Diet Plus showed significantly higher concentrations of Thr and His compared to the other treatments, while Phe and Lys showed significantly higher concentrations compared to the non-selected strain fed Diet Minus (Fig. 6a,c). Also, at 3 h the selected strain fed Diet Plus showed significantly higher concentration for Leu compared either to the selected and non-selected strains fed Diet Minus. At 6 h the non-selected strain fed Diet Minus showed significantly higher Leu concentration compared to the selected strain fed Diet Minus and to the non-selected strain fed Diet Plus. At 12 h the non-selected strain showed significantly higher Thr concentration compared to the other groups while the selected strain fed Diet Minus showed higher Leu concentration compared to the non-selected fed Diet Minus. At 24 h the non-selected strain fed Diet Plus showed significantly higher Lys concentration compared to the other groups. A strain by time significant interaction (P < 0.05) was detected for plasma concentrations of Val and Met. At 3 h, the selected strain showed a significantly higher concentration of Met compared to the non-selected strain. At 18 h the non-selected strain showed significantly higher plasma concentration for Val compared to the selected strain. A diet by time significant effect (P < 0.05) was detected for Val, at 3 h post-prandially in both fish strains fed Diet Plus compared to fish fed Diet Minus. Time had a significant effect (P < 0.01) on Arg and Ile plasma concentrations. At 24 h post-prandially, both amino acids reached the lowest concentrations (Fig. 7a,c). Finally, a significant diet (P < 0.01) effect was observed regarding Met and Arg plasma concentrations with the fish fed Diet Plus showing significantly higher concentrations compared fish fed Diet Minus.

In the CV, a significant diet by strain by time interaction (P < 0.05) was detected for Thr, Val, Met, Ile, Leu, His and Lys plasma concentrations (Table S12). At 3 h the selected strain fed Diet Plus showed significantly higher concentrations of Thr and His compared to the other treatments, and Lys was higher compared to the selected and non-selected groups fed Diet Minus (Fig. 6b,d). At 6 h the selected strain fed Diet Minus showed lower Leu concentration compared to the non-selected strain. At 6 h the selected strain fed Diet Plus had higher concentration of Lys compared to the selected strain fed Diet Minus. At 18 h the non-selected strain fed Diet Plus showed significantly higher plasma concentrations of Thr and Lys compared to all the other groups, while Ile plasma concentration was higher compared to the selected strain fed Diet Plus (Fig. 7b,d). At 24 h the non-selected strain fed Diet Plus showed significantly higher concentrations for Thr and Lys compared to the other groups and for His, Val and Ile compared to the selected strain fed Diet Minus. For Leu, the selected strain fed Diet Minus showed significantly lower values compared to the non-selected strain fed either Diet Plus or Diet Minus. Time had a significant effect (P < 0.01) on Arg plasma concentration with the 24 h being significantly lower than the 6 and 3 h. A diet effect also was detected for Arg with the fish fed Diet Plus showing significantly higher plasma concentrations compared to fish fed Diet Minus (P < 0.001).

## Discussion

To our knowledge this is the first study to explore and provide novel insights into the physiological mechanisms that allow a carnivorous fish species to thrive when fed an all-plant protein diet. Apparent digestibility coefficient (ADC) results demonstrate that selection for improved growth and plant protein utilization in rainbow trout does not affect apparent digestibility of nutrients. Our results showed a diet effect with the all-plant protein-based diet having higher protein digestibility which is in accordance with other studies^19,20^. Callet *et al*.^20^ compared a rainbow trout strain after three generations of selection on a plant-based diet with a control line strain in a 2×2 factorial design (strain by diet) and did not detect any interaction. However, they found a significant increase in ADC of protein and decreased values for energy, lipid, moisture and starch when fish were fed an all plant-based diet compared to a fishmeal-based diet. Although measuring apparent digestibility of nutrients remains an important tool for evaluating feed ingredient quality, it cannot be considered sufficient to assess metabolic utilization of amino acids because it does not provide information regarding specific rates of nutrient absorption and metabolism^21^.

Concentrations of plasma amino acids collected in the HPV were elevated compared to the systemic blood amino acid concentrations levels found in samples from the CV. In a study conducted by Karlsson *et al*.^21^ using cannulated rainbow trout and force-fed 1% body weight, similar differences in amino acid concentrations between HPV and dorsal aorta samples were found. These authors postulated that blood returning to the sinus venosus from the hepatic circulation is diluted by other systemic venous return in direct proportion to the relative proportion of hepatic blood flow^21^. In the present study, plasma amino acid profiles were strongly affected by dietary source and reflected the amino acid composition of ingredients while also maintaining their relative ratios over time. This agrees with other studies on rainbow trout^14,22–25^. We found significant interactions of strain by time in all the tested ingredients in the HPV, except for soy protein concentrate. In contrast, in the CV there was no interaction found across ingredients, except for valine in wheat gluten meal. Plasma amino acid measurements from the CV provides less resolution regarding protein digestion rates compared to the HPV and this may be related to hepatic and post-hepatic metabolism in contrast to intestinal uptake^21^.

Fishmeal is defatted, dried meal and the protein source of choice in feeds because carnivorous fish are piscivorous. Thus, the postprandrial pattern of plasma amino acids in fish fed fishmeal is likely to closely match that of fish consuming natural prey. The fish force-fed fishmeal showed a peak in the HPV for all the plasma amino acids at 12 h postprandial, with the selected strain reaching higher levels compared to the non-selected strain, while in the CV the plasma amino acids peaked at 18 h. Fishmeal, as expected, showed an overall homogeneous pattern for all the amino acids, similar to findings in other studies^14^. Replacing dietary fishmeal with plant protein concentrates results in a shift in postprandial plasma amino acid temporal profiles and synchronization^14,23^. This shift is assumed to be caused by antinutritional factors, protein solubility differences, and gastric evacuation rate differences which ultimately affect the digestion rate of plant proteins^12,24,26^. In our study, the non-selected strain showed marked differences in plasma amino acid concentrations when force-fed plant protein concentrates compared to fishmeal. Comparing strains fed individual protein concentrates, the selected strain showed higher peaks in the HPV at 12 h postprandially when force-fed either soybean meal or wheat gluten meal compared to the non-selected strain which showed a later peak when fed soybean at 18 h and an earlier peak at 6 h when force-fed wheat gluten meal. In the CV, plasma amino acid concentrations did not differ significantly for the two strains fed either ingredient. These results suggest that selection has altered the temporal dynamics of plant protein digestion and absorption, thus providing an explanation for the higher growth and protein retention observed in the selected strain fed plant protein-based diets. Wheat gluten was the only ingredient for which both the non-selected and selected strains exhibited similar amino acid profiles at 12 h postprandially regarding the caudal vein. Wheat gluten meal postprandial amino acid patterns for the selected strain were homogeneous and similar to fishmeal patterns, except for lysine content, which was lower and in agreement with previous studies^23,27^. Not surprisingly, wheat gluten is considered comparable to fishmeal when supplemented with amino acids and research has shown that wheat gluten can replace fishmeal in rainbow trout diets^28^. An interaction between strain and time for threonine and leucine was detected in the HPV of fish fed corn protein concentrate with the selected strain showing peaks at 18 h postprandially. Other amino acids, although not significantly different between strains, were higher in concentration at 18 h in the selected strain in the HPV. In contrast, in the CV, the non-selected strain showed significantly higher plasma amino acid concentrations compared to the selected strain. For corn protein concentrate the concentrations of most of the plasma amino acids measured in the hepatic or caudal veins were lower compared to the other plant protein ingredients with the exception of soybean meal in the caudal vein, despite the fact that the protein content of corn protein concentrate is relatively high (75%). Finally, soy protein concentrate was the only ingredient for which interactions were not found and further, no strain effect was detected. The only significant effect found was related to time with two major peaks found in the HPV at 6 and 18 h postprandial, while in the CV a plateau for almost all the amino acids was observed between 6 and 18 h. Soy protein concentrate is considered one of the most promising plant protein sources to replace fishmeal due to its high protein content and lower antinutritional factor levels. Several studies have reported that high inclusion levels showed comparable results to a fishmeal-based diets^29–32^.

No significant interactions were found between the selected and non-selected strains when fed amino acid supplemented or non-supplemented protein concentrate mixtures. In the HPV, balancing the plant protein mixture with supplemental amino acids (Diet Plus) not only increased concentrations of all the essential amino acids but notably had an effect on plasma amino acids temporal behavior. The selected strain fed Diet Plus showed a peak in amino acid uptake at 3 h postprandially in contrast to the other treatments. However, supplementation with amino acids generally led to an alteration of all dietary essential amino acid uptake in both strains compared to the non-supplemented mixture. Moreover, the selected strain fed Diet Plus showed a noteworthy difference compared to the non-selected strain, specifically, a synchronous and homogenous decreasing pattern for all the essential amino acids over time. Further, significant interactions were detected in CV samples for most of the plasma amino acids with the selected strain maintaining the same synchronized plasma amino acid decreasing pattern as was showed in the HPV. In contrast, the non-selected strain showed significantly higher concentrations at 24 h postprandially for arginine, threonine, valine, leucine and very high concentration of lysine compared to the other treatments. The interactions found in the CV demonstrate the strong effect that an all-plant protein mixture can have on the digestive physiology of a carnivorous fish species. Studies using rainbow trout showed that feeding a plant-protein mixture leads to much less synchronous amino acid uptake compared to when fishmeal is replaced by a single plant protein source, suggesting that different plant-based protein ingredients are diverse in the way they affect the uptake of dietary amino acids^14^. Research in swine has demonstrated asynchronous nutrient absorption patterns can be induced by formulating diets using ingredients with different digestion and absorption kinetics^33^. Furthermore, in the present study, the addition of crystalline amino acids into the all-plant protein mixture affected the plasma concentrations of all amino acids as it did for uptake reflected in the HPV. Rolland *et al*.^34^ showed that supplementing a diet with methionine as a single amino acid influenced the plasma profiles and concentrations of other essential amino acids. However, in the present study the selected strain showed a remarkably synchronized dietary amino acid uptake pattern which influenced the pattern of postprandial appearance of free amino acids in the systemic blood over time. We assume that the fast and homogeneous dietary amino acid uptake in the HPV and the fast-postprandial plasma amino acid disappearance are results of selection for growth on and tolerance of an all-plant protein diet. The selected strain has more rapid growth (~10%) and higher protein retention efficiency (~15%) when fed an all-plant protein diet compared to a non-selected rainbow trout strain fed a fishmeal-based diet^15^. For optimal amino acid utilization to occur, postprandial plasma amino acid appearance rates should not exceed net protein synthesis capacity. Compared to mammals, the amino acid pool in fish available for protein synthesis derived from intracellular protein degradation is much less^35^; implying that a transient amino acid imbalance would have negative effects on muscle protein turnover. We hypothesize that plasma amino acid synchronization and increased genetic potential for growth of the selected strain can explain the postprandial plasma amino acid disappearance rate. Our findings are in accordance with a study in growing pigs which was designed to evaluate the effects of synchronized amino acid availability on protein metabolism^33^. The researchers reported a reduction in protein retention from 57% to 47% in pigs fed a balanced diet characterized by asynchronous temporally amino acid availability.

## Conclusion

This is the first study to explore and provide novel insights on digestive physiology of a carnivorous fish strain genetically selected over six generations for improved plant protein utilization efficiency and growth. Our findings demonstrated that improved performance of the selected strain is associated with a synchronous protein digestion of the plant protein mixture and synchronization of amino acid absorption leading to improved availability and utilization. Protein and amino acid digestibility, though a useful quality assessment tool, does not provide information related to temporal nutrient absorption. In contrast, monitoring temporal plasma amino acid patterns allows assessment of absorption rates and overall metabolic utilization of amino acids. However, temporal plasma amino acid patterns of single ingredients cannot be used to predict amino acid dynamics when ingredients are combined in a blend. Most importantly, the results showed that supplementation of amino acids to a plant protein concentrate mixture affects the digestion process of the diet in terms of uptake and utilization of all essential amino acids. These insights were made possible by using the selected rainbow trout strain as a unique model to pursue the discovery of physiological mechanisms associated with increasing use of plant proteins in sustainable fish feeds.

## Supplementary information

Supplementary Tables.
