# Supplementary material for: Improved performance of a rainbow trout selected strain is associated with protein digestion rates and synchronization of amino acid absorption
Source: Sci Rep. 2020 Mar 13;10:4678. doi: 10.1038/s41598-020-61360-0 (PMC7069933; doi:10.1038/s41598-020-61360-0)
Supplement: Supplementary file 1 — Supplementary Tables. [file 41598_2020_61360_MOESM1_ESM.docx]

**Improved performance of a rainbow trout selected strain is associated with protein digestion rates and synchronization of amino acid absorption**

Andreas Brezas and Ronald W. Hardy*

Hagerman Fish Culture Experiment Station, University of Idaho, 3059F National Fish Hatchery Road, Hagerman, ID 83332, USA

*** Corresponding author:** [rhardy@uidaho.edu](mailto:rhardy@uidaho.edu)

**Supplementary tables**

**Table S1.** Free essential amino acid (except tryptophan) mean concentrations n=3 ± SEM in blood plasma (nmol/mL) collected from the hepatic portal vein of two strains of rainbow trout during a 24h period after force feeding of fishmeal. When interaction is present no superscripts are assigned in main factors.

| STRAIN | | Thr | Val | Met | Ile | Leu | Phe | His | Lys | Arg |
| --- | --- | --- | --- | --- | --- | --- | --- | --- | --- | --- |
| NON SEL | | 420±36 | 576±35 | 153±13 | 249±22a | 438±38 | 123±1 | 194±14 | 509±61 | 266±36 |
| SEL | | 437±54 | 636±78 | 148±19 | 305±46b | 523±76 | 137±16 | 196±17 | 554±83 | 286±47 |
| P-value | | ns | ns | ns | P<0.05 | ns | ns | ns | ns | ns |
|  | |  |  |  |  |  |  |  |  |  |
| TIME | | **Thr** | **Val** | **Met** | **Ile** | **Leu** | **Phe** | **His** | **Lys** | **Arg** |
| 3H | | 277±49^a^ | 414±46 | 103±18 | 180±31 | 328±59 | 110±19^a^ | 167±23^a^ | 438±105^a^ | 251±65^a^ |
| 6H | | 387±37^ab^ | 536±36 | 139±12 | 253±18 | 450±32 | 128±10^a^ | 187±10^a^ | 560±48^a^ | 296±34^a^ |
| 12H | | 684±101^c^ | 933±153 | 228±29 | 484±94 | 827±148 | 213±32^b^ | 292±12^b^ | 987±93^b^ | 520±49^b^ |
| 18H | | 485±43^b^ | 732±52 | 187±9 | 335±37 | 580±59 | 130±13^a^ | 191±22^a^ | 507±83^a^ | 266±45^a^ |
| 24H | | 869±37^ab^ | 497±47 | 112±24 | 190±24 | 315±37 | 96±10^a^ | 167±16^a^ | 305±46^a^ | 127±28^a^ |
| P-value | | P<0.001 | P<0.001 | P<0.001 | P<0.001 | P<0.001 | P<0.01 | P<0.01 | P<0.001 | P<0.001 |
|  | |  |  |  |  |  |  |  |  |  |
| STRAIN x TIME | | **Thr** | **Val** | **Met** | **Ile** | **Leu** | **Phe** | **His** | **Lys** | **Arg** |
| NON SEL | 3H | 251±55 | 372±50^a^ | 82±11^ab^ | 146±44^a^ | 270±78^a^ | 91±29 | 143±41 | 309±178 | 1170±107 |
| NON SEL | 6H | 425±65 | 576±55^abc^ | 151±11^ab^ | 257±39^ab^ | 470±65^abc^ | 132±19 | 194±13 | 611±83 | 338±57 |
| NON SEL | 12H | 536±117 | 710±33^bc^ | 183±26^b^ | 347±31^ab^ | 614±45^bc^ | 172±25 | 280±15 | 852±23 | 450±14 |
| NON SEL | 18H | 459±76 | 631±55^abc^ | 185±19^b^ | 269±38^ab^ | 475±68^abc^ | 117±20 | 182±22 | 434±95 | 234±51 |
| NON SEL | 24H | 411±57 | 568±61^abc^ | 148±38^ab^ | 225±35^ab^ | 365±53^abc^ | 110±18 | 185±25 | 386±55 | 169±43 |
| SEL | 3H | 294±82 | 442±71^ab^ | 116±28^ab^ | 202±43^ab^ | 367±87^abc^ | 122±26 | 183±29 | 524±129 | 305±82 |
| SEL | 6H | 350±35 | 495±43^ab^ | 127±20^ab^ | 248±14^ab^ | 429±21^abc^ | 125±13 | 180±16 | 509±42 | 254±27 |
| SEL | 12H | 833±53 | 1155±203^d^ | 273±15^c^ | 621±122^c^ | 1040±197^d^ | 253±46 | 305±18 | 1122±123 | 590±65 |
| SEL | 18H | 510±53 | 833±20^c^ | 188±6^b^ | 401±34^b^ | 686±43^c^ | 142±18 | 201±43 | 580±142 | 298±82 |
| SEL | 24H | 328±45 | 427±50^ab^ | 75±14^a^ | 156±19^a^ | 265±40^ab^ | 83±6 | 149±17 | 224±33 | 85±19 |
| P-value |  | ns | P<0.01 | P<0.05 | P<0.05 | P<0.05 | ns | ns | ns | ns |

**Table S2.** Free essential amino acid (except tryptophan) mean concentrations n=3 ± SEM in blood plasma (nmol/mL) collected from the caudal vein of two strains of rainbow trout during a 24h period after force feeding of fishmeal. When interaction is present no superscripts are assigned in main factors.

| STRAIN | | Thr | Val | Met | Ile | Leu | Phe | His | Lys | Arg |
| --- | --- | --- | --- | --- | --- | --- | --- | --- | --- | --- |
| NON SEL | | 349±28 | 516±28 | 156±13 | 220±16 | 358±27 | 97±5 | 174±6^b^ | 348±31 | 190±20 |
| SEL | | 335±25 | 559±39 | 143±11 | 255±22 | 415±36 | 106±5 | 152±6^a^ | 398±35 | 196±18 |
| P-value | | ns | ns | ns | ns | ns | ns | P<0.05 | ns | ns |
|  | |  |  |  |  |  |  |  |  |  |
| TIME | | **Thr** | **Val** | **Met** | **Ile** | **Leu** | **Phe** | **His** | **Lys** | **Arg** |
| 3H | | 244±17^a^ | 411±24^a^ | 106±5^a^ | 180±12^a^ | 287±20^a^ | 100±3^b^ | 156±6^ab^ | 311±16^ab^ | 169±8^bc^ |
| 6H | | 346±42^ab^ | 528±28^b^ | 153±14^abc^ | 248±20^b^ | 408±31^b^ | 118±5^b^ | 170±11^ab^ | 436±32^cd^ | 253±25 ^de^ |
| 12H | | 400±45^b^ | 624±34^c^ | 170±14^bc^ | 284±21^bc^ | 461±33^bc^ | 107±5^b^ | 178±6^b^ | 508±42^d^ | 246±18^e^ |
| 18H | | 406±15^b^ | 710±34^c^ | 188±14^c^ | 327±23^c^ | 539±35^c^ | 111±6^b^ | 170±8^b^ | 387±34^bc^ | 194±11^cd^ |
| 24H | | 305±32^ab^ | 430±25^a^ | 127±21^a^ | 159±10^a^ | 256±20^a^ | 74±4^a^ | 137±12^a^ | 222±30^a^ | 99±15^a^ |
| P-value | | P<0.05 | P<0.001 | P<0.01 | P<0.001 | P<0.001 | P<0.001 | P<0.05 | P<0.001 | P<0.001 |
|  | |  |  |  |  |  |  |  |  |  |
| STRAIN x TIME | | **Thr** | **Val** | **Met** | **Ile** | **Leu** | **Phe** | **His** | **Lys** | **Arg** |
| NON SEL | 3H | 257±26 | 426±15 | 112±10 | 185±13 | 300±34 | 102±7 | 169±6 | 309±34 | 178±16 |
| NON SEL | 6H | 353±85 | 526±54 | 151±26 | 240±40 | 393±64 | 115±9 | 181±20 | 423±67 | 260±53 |
| NON SEL | 12H | 383±64 | 580±40 | 164±24 | 250±18 | 404±26 | 98±6 | 182±8 | 440±58 | 222±28 |
| NON SEL | 18H | 400±31 | 641±4 | 218±11 | 275±1 | 461±4 | 103±8 | 181±18 | 324±23 | 176±22 |
| NON SEL | 24H | 340±61 | 420±44 | 139±32 | 155±17 | 249±30 | 73±7 | 158±16 | 222±24 | 104±16 |
| SEL | 3H | 235±26 | 402±41 | 103±7 | 176±20 | 278±29 | 99±4 | 148±5 | 313±21 | 164±8 |
| SEL | 6H | 339±42 | 531±33 | 154±15 | 256±20 | 423±24 | 120±4 | 160±9 | 449±22 | 247±13 |
| SEL | 12H | 417±75 | 668±48 | 176±20 | 317±28 | 518±40 | 116±6 | 174±8 | 576±26 | 270±17 |
| SEL | 18H | 411±21 | 756±34 | 168±11 | 362±15 | 591±28 | 117±7 | 162±7 | 429±38 | 206±10 |
| SEL | 24H | 271±16 | 440±35 | 115±32 | 162±14 | 264±31 | 76±4 | 116±3 | 223±63 | 94±29 |
| P-value | | ns | ns | ns | ns | ns | ns | ns | ns | ns |

**Table S3.** Free essential amino acid (except tryptophan) mean concentrations n=3 ± SEM in blood plasma (nmol/mL) collected from the hepatic portal vein of two strains of rainbow trout during a 24h period after force feeding of soy protein concentrate. When interaction is present no superscripts are assigned in main factors.

| STRAIN | | Thr | Val | Met | Ile | Leu | Phe | His | Lys | Arg |
| --- | --- | --- | --- | --- | --- | --- | --- | --- | --- | --- |
| NON SEL | | 336±23 | 532±30 | 67±5 | 262±20 | 440±32 | 140±9 | 178±13 | 416±34 | 314±34 |
| SEL | | 312±16 | 498±25 | 65±6 | 246±18 | 407±28 | 131±9 | 168±9 | 387±26 | 290±27 |
| P-value | | ns | ns | ns | ns | ns | ns | ns | ns | ns |
|  | |  |  |  |  |  |  |  |  |  |
| TIME | | **Thr** | **Val** | **Met** | **Ile** | **Leu** | **Phe** | **His** | **Lys** | **Arg** |
| 3H | | 283±12^a^ | 470±31^a^ | 79±4^cd^ | 231±17^ab^ | 381±27^ab^ | 150±4^b^ | 199±13^b^ | 397±6^b^ | 315±11^b^ |
| 6H | | 405±10^b^ | 616±29^b^ | 88±4^d^ | 345±20^c^ | 557±30^c^ | 185±3^c^ | 205±7^b^ | 585±25^c^ | 494±23^c^ |
| 12H | | 311±17^a^ | 507±28^ab^ | 64±4^b^ | 252±19^b^ | 437±33^b^ | 127±9^b^ | 156±9^ab^ | 419±36^b^ | 305±33^b^ |
| 18H | | 391±28^b^ | 594±43^b^ | 68±5^bc^ | 294±27^bc^ | 487±43^bc^ | 136±12^b^ | 187±18^b^ | 411±29^b^ | 305±24^b^ |
| 24H | | 256±21^a^ | 424±24^a^ | 39±2^a^ | 178±14^a^ | 304±21^a^ | 97±6^a^ | 126±7^a^ | 260±17^a^ | 158±16^a^ |
| P-value | | P<0.001 | P<0.01 | P<0.001 | P<0.001 | P<0.001 | P<0.001 | P<0.001 | P<0.001 | P<0.001 |
|  | |  |  |  |  |  |  |  |  |  |
| STRAIN x TIME | | **Thr** | **Val** | **Met** | **Ile** | **Leu** | **Phe** | **His** | **Lys** | **Arg** |
| NON SEL | 3H | 282±20 | 518±51 | 72±3 | 254±28 | 419±48 | 152±7 | 200±24 | 392±12 | 306±21 |
| NON SEL | 6H | 409±19 | 608±69 | 86±9 | 337±46 | 547±69 | 187±4 | 215±5 | 606±47 | 524±14 |
| NON SEL | 12H | 334±17 | 519±46 | 67±5 | 265±31 | 460±52 | 139±8 | 162±10 | 451±50 | 341±35 |
| NON SEL | 18H | 441±37 | 656±21 | 79±2 | 324±10 | 539±18 | 151±9 | 215±23 | 461±20 | 345±21 |
| NON SEL | 24H | 236±23 | 387±26 | 36±1 | 154±13 | 271±21 | 88±6 | 113±5 | 231±19 | 124±8 |
| SEL | 3H | 283±16 | 423±6 | 86±4 | 207±2 | 343±3 | 147±3 | 199±18 | 402±5 | 323±9 |
| SEL | 6H | 402±15 | 624±13 | 90±3 | 352±14 | 568±22 | 182±6 | 194±6 | 564±24 | 464±34 |
| SEL | 12H | 275±7 | 489±33 | 58±5 | 234±12 | 403±22 | 109±4 | 147±19 | 371±40 | 249±45 |
| SEL | 18H | 341±14 | 532±70 | 57±1 | 265±53 | 435±80 | 122±21 | 160±19 | 362±36 | 266±29 |
| SEL | 24H | 276±34 | 460±30 | 41±4 | 202±16 | 338±25 | 106±6 | 140±4 | 289±15 | 191±9 |
| P-value | | ns | ns | ns | ns | ns | ns | ns | ns | ns |

**Table S4.** Free essential amino acid (except tryptophan) mean concentrations n=3 ± SEM in blood plasma (nmol/mL) collected from the caudal vein of two strains of rainbow trout during a 24h period after force feeding of soy protein concentrate. When interaction is present no superscripts are assigned in main factors.

| STRAIN | | Thr | | Val | | Met | | Ile | Leu | | Phe | | His | | Lys | | Arg | |  |
| --- | --- | --- | --- | --- | --- | --- | --- | --- | --- | --- | --- | --- | --- | --- | --- | --- | --- | --- | --- |
| NON SEL | | 287±22 | | 489±28 | | 69±5 | | 231±17 | 363±26 | | 112±6 | | 174±18 | | 305±23 | | 237±25 | |  |
| SEL | | 300±17 | | 495±24 | | 74±7 | | 243±15 | 377±23 | | 115±5 | | 169±9 | | 352±20 | | 257±16 | |  |
| P-value | | ns | | ns | | ns | | ns | ns | | ns | | ns | | ns | | ns | |  |
|  | |  | |  | |  | |  |  | |  | |  | |  | |  | |  |
| TIME | | **Thr** | | **Val** | | **Met** | | **Ile** | **Leu** | | **Phe** | | **His** | | **Lys** | | **Arg** | |  |
| 3H | | 245±28^ab^ | | 441±28^ab^ | | 87±10^b^ | | 203±13^ab^ | 318±25^ab^ | | 127±7^b^ | | 220±36 | | 323±41^b^ | | 220±27 | |  |
| 6H | | 340±19^c^ | | 514±23^b^ | | 82±6^b^ | | 255±15^b^ | 396±23^b^ | | 125±6^b^ | | 169±7 | | 387±9^b^ | | 336±12 | |  |
| 12H | | 313±14^bc^ | | 546±34^b^ | | 79±3^b^ | | 276±21^b^ | 428±32^b^ | | 110±6^b^ | | 173±8 | | 381±23^b^ | | 272±23 | |  |
| 18H | | 345±28^c^ | | 554±46^b^ | | 65±7^b^ | | 272±27^b^ | 424±40^b^ | | 112±10^ab^ | | 172±18 | | 317±23^b^ | | 254±17 | |  |
| 24H | | 209±25^a^ | | 388±29^a^ | | 40±3^a^ | | 168±18^a^ | 265±25^a^ | | 89±7^a^ | | 113±6 | | 212±23^a^ | | 133±22 | |  |
| P-value | | P<0.01 | | P<0.05 | | P<0.001 | | P<0.01 | P<0.05 | | P<0.05 | | ns | | P<0.01 | | P<0.001 | |  |
|  | |  | |  | |  | |  |  | |  | |  | |  | |  | |  |
| STRAIN x TIME | | **Thr** | | **Val** | | **Met** | | **Ile** | **Leu** | | **Phe** | | **His** | | **Lys** | | **Arg** | |  |
| NON SEL | 3H | | 203±9 | | 442±39 | | 72±14 | 195±13 | | 302±27 | | 119±9 | | 229±78 | | 251±10 | | 169±14^ab^ | |
| NON SEL | 6H | | 347±12 | | 528±43 | | 82±12 | 263±28 | | 409±42 | | 134±9 | | 181±8 | | 385±3 | | 348±20^c^ | |
| NON SEL | 12H | | 307±19 | | 514±54 | | 75±4 | 260±35 | | 410±56 | | 109±2 | | 161±9 | | 378±46 | | 287±32^c^ | |
| NON SEL | 18H | | 381±40 | | 595±65 | | 76±9 | 284±36 | | 449±53 | | 114±17 | | 193±32 | | 320±47 | | 274±26^bc^ | |
| NON SEL | 24H | | 196±27 | | 367±33 | | 41±3 | 153±18 | | 245±28 | | 85±8 | | 107±6 | | 193±23 | | 107±19^a^ | |
| SEL | 3H | | 288±46 | | 440±48 | | 101±10 | 211±26 | | 334±47 | | 136±9 | | 211±19 | | 395±57 | | 270±28^bc^ | |
| SEL | 6H | | 332±40 | | 500±26 | | 82±6 | 246±16 | | 382±25 | | 116±2 | | 157±4 | | 389±19 | | 324±15^c^ | |
| SEL | 12H | | 318±23 | | 579±43 | | 83±4 | 291±27 | | 447±39 | | 112±12 | | 185±7 | | 384±25 | | 257±37^b^ | |
| SEL | 18H | | 309±32 | | 512±69 | | 53±5 | 260±48 | | 400±69 | | 110±13 | | 151±8 | | 314±19 | | 234±19^b^ | |
| SEL | 24H | | 229±60 | | 419±58 | | 38±7 | 191±36 | | 295±49 | | 95±16 | | 124±5 | | 240±47 | | 173±31^ab^ | |
| P-value | | ns | | ns | | ns | | ns | ns | | ns | | ns | | ns | | P<0.05 | |  |

**Table S5**. Free essential amino acid (except tryptophan) mean concentrations n=3 ± SEM in blood plasma (nmol/mL) collected from the hepatic portal vein of two strains of rainbow trout during a 24h period after force feeding of soybean meal. When interaction is present no superscripts are assigned in main factors.

| STRAIN | Thr | | Val | | Met | | Ile | | Leu | | Phe | | His | | Lys | | Arg |
| --- | --- | --- | --- | --- | --- | --- | --- | --- | --- | --- | --- | --- | --- | --- | --- | --- | --- |
| NON SEL | 283±12 | | 443±14 | | 60±2 | | 219±10 | | 339±15 | | 134±6 | | 151±6 | | 373±19 | | 239±20 |
| SEL | 276±12 | | 423±27 | | 56±2 | | 227±20 | | 339±29 | | 143±10 | | 151±7 | | 347±21 | | 270±22 |
| P-value | **ns** | | **ns** | | **ns** | | **ns** | | **ns** | | **ns** | | **ns** | | **ns** | | **P<0.05** |
|  |  | |  | |  | |  | |  | |  | |  | |  | |  |
| TIME | **Thr** | | **Val** | | **Met** | | **Ile** | | **Leu** | | **Phe** | | **His** | | **Lys** | | **Arg** |
| 3H | 289±31 | | 407±26 | | 61±3^b^ | | 185±14 | | 288±22 | | 112±4 | | 133±6 | | 363±27 | | 194±20 |
| 6H | 272±13 | | 379±34 | | 63±3^b^ | | 183±16 | | 275±26 | | 125±7 | | 151±7 | | 335±30 | | 229±19 |
| 12H | 311±11 | | 515±33 | | 64±2^b^ | | 284±28 | | 429±40 | | 175±15 | | 172±11 | | 399±37 | | 333±40 |
| 18H | 276±18 | | 470±27 | | 53±4^ab^ | | 266±17 | | 400±26 | | 159±10 | | 159±10 | | 389±38 | | 310±29 |
| 24H | 251±13 | | 395±20 | | 49±3^a^ | | 197±11 | | 302±17 | | 124±5 | | 141±6 | | 314±16 | | 207±9 |
| P-value | ns | | P<0.01 | | P<0.01 | | P<0.001 | | P<0.001 | | P<0.001 | | P<0.001 | | P<0.05 | | P<0.001 |
|  |  | |  | |  | |  | |  | |  | |  | |  | |  |
| STRAIN x TIME |  | | **Thr Val** | | **Met** | | **Ile** | | **Leu** | | **Phe** | | **His** | | **Lys** | | **Arg** |
| NON SEL | 3H | 288±51 | | 427±34^ab^ | | 64±6 | | 187±20^a^ | | 293±29^ab^ | | 114±8^a^ | | 127±8^a^ | | 395±43^ab^ | 155±20^a^ |
| NON SEL | 6H | 283±10 | | 446±19^ab^ | | 68±2 | | 210±11^ab^ | | 422±20^ab^ | | 125±8^a^ | | 165±5^abc^ | | 389±23^ab^ | 236±29^a^ |
| NON SEL | 12H | 321±4 | | 452±18^ab^ | | 64±1 | | 226±7^ab^ | | 349±14^ab^ | | 142±1^ab^ | | 151±4^ab^ | | 325±6^a^ | 250±8^a^ |
| NON SEL | 18H | 292±12 | | 507±16^bc^ | | 60±3 | | 282±7^b^ | | 430±11^bc^ | | 173±6^b^ | | 178±6^bc^ | | 461±23^b^ | 360±16^b^ |
| NON SEL | 24H | 233±20 | | 383±30^ab^ | | 47±4 | | 190±13^a^ | | 299±23^ab^ | | 118±5^a^ | | 134±7^a^ | | 297±27^a^ | 193±7^a^ |
| SEL | 3H | 291±46 | | 387±43^ab^ | | 59±2 | | 183±25^a^ | | 282±39^a^ | | 110±5^a^ | | 138±10^a^ | | 332±26^a^ | 232±14^a^ |
| SEL | 6H | 261±25 | | 313±32^a^ | | 59±5 | | 156±21^a^ | | 229±31^a^ | | 124±14^a^ | | 136±6^a^ | | 281±32^a^ | 223±29^a^ |
| SEL | 12H | 300±21 | | 577±32^c^ | | 65±5 | | 342±24^c^ | | 510±36^c^ | | 207±11^c^ | | 194±12^c^ | | 473±37^b^ | 416±32^b^ |
| SEL | 18H | 260±35 | | 4 33±44^ab^ | | 46±6 | | 249±35^ab^ | | 369±49^ab^ | | 145±16^a^ | | 140±12^a^ | | 318±41^a^ | 261±37^a^ |
| SEL | 24H | 268±13 | | 407±30^ab^ | | 51±4 | | 204±19^ab^ | | 306±30^ab^ | | 129±7^a^ | | 148±8^ab^ | | 331±17^a^ | 221±14^a^ |
| P-value | ns | | P<0.01 | | ns | | P<0.01 | | P<0.01 | | P<0.001 | | P<0.001 | | P<0.001 | | P<0.001 |

**Table S6.** Free essential amino acid (except tryptophan) mean concentrations n=3 ± SEM in blood plasma (nmol/mL) collected from the caudal vein of two strains of rainbow trout during a 24h period after force feeding of soybean meal. When interaction is present no superscripts are assigned in main factors.

| STRAIN | | Thr | Val | Met | Ile | Leu | Phe | His | Lys | Arg |
| --- | --- | --- | --- | --- | --- | --- | --- | --- | --- | --- |
| NON SEL | | 192±8 | 361±12 | 51±3 | 158±7 | 246±12 | 99±5 | 122±5 | 237±16 | 146±11 |
| SEL | | 181±22 | 355±24 | 46±3 | 168±14 | 251±20 | 103±5 | 112±6 | 220±21 | 160±12 |
| P-value | | ns | ns | ns | ns | ns | ns | ns | ns | ns |
|  | |  |  |  |  |  |  |  |  |  |
| TIME | | **Thr** | **Val** | **Met** | **Ile** | **Leu** | **Phe** | **His** | **Lys** | **Arg** |
| 3H | | 262±36 | 403±40 | 64±5^b^ | 168±23^ab^ | 256±33^ab^ | 87±4^a^ | 125±11 | 260±44 | 128±15^ab^ |
| 6H | | 170±14 | 292±34 | 50±3^a^ | 118±15^a^ | 181±25^a^ | 83±7^a^ | 102±8 | 183±26 | 111±17^a^ |
| 12H | | 181±12 | 369±21 | 46±4^a^ | 174±14^ab^ | 268±21^b^ | 112±6^b^ | 121±8 | 208±9 | 172±9^b^ |
| 18H | | 175±11 | 386±11 | 41±4^a^ | 196±9^b^ | 295±12^b^ | 115±4^b^ | 118±12 | 237±21 | 177±16^b^ |
| 24H | | 157±37 | 345±24 | 44±3^a^ | 160±11^ab^ | 244±17^ab^ | 105±4^b^ | 121±8 | 257±36 | 173±18^b^ |
| P-value | | ns | ns | P<0.01 | P<0.05 | P<0.05 | P<0.001 | ns | ns | P<0.05 |
|  | |  |  |  |  |  |  |  |  |  |
| STRAIN x TIME | | **Thr** | **Val** | **Met** | **Ile** | **Leu** | **Phe** | **His** | **Lys** | **Arg** |
| NON SEL | 3H | 220±53 | 365±24 | 62±11 | 142±24 | 219±32 | 82±1 | 112±3 | 259±103 | 112±3 |
| NON SEL | 6H | 190±20 | 357±23 | 56±2 | 144±9 | 223±16 | 81±9 | 115±12 | 223±34 | 118±33 |
| NON SEL | 12H | 203±1 | 347±39 | 54±4 | 149±17 | 237±35 | 106±6 | 121±18 | 195±12 | 157±9 |
| NON SEL | 18H | 179±7 | 387±4 | 43±3 | 185±1 | 288±1 | 119±5 | 142±10 | 268±15 | 177±27 |
| NON SEL | 24H | 177±11 | 349±42 | 43±5 | 162±19 | 254±34 | 102±4 | 116±5 | 247±33 | 154±12 |
| SEL | 3H | 290±49 | 429±66 | 66±6 | 185±34 | 280±50 | 91±5 | 133±17 | 261±53 | 139±24 |
| SEL | 6H | 151±13 | 228±33 | 43±4 | 92±19 | 138±31 | 85±14 | 88±4 | 143±23 | 104±18 |
| SEL | 12H | 158±15 | 390±16 | 38±3 | 199±7 | 299±9 | 118±9 | 120±2 | 221±9 | 187±6 |
| SEL | 18H | 172±24 | 386±23 | 39±8 | 207±16 | 302±25 | 111±7 | 94±1 | 207±32 | 177±24 |
| SEL | 24H | 137±79 | 341±34 | 44±6 | 157±14 | 234±17 | 109±7 | 126±17 | 267±74 | 191±34 |
| P-value | | ns | ns | ns | ns | ns | ns | ns | ns | ns |

**Table S7.** Free essential amino acid (except tryptophan) mean concentrations n=3 ± SEM in blood plasma (nmol/mL) collected from the hepatic portal vein of two strains of rainbow trout during a 24h period after force feeding of corn protein concentrate. When interaction is present no superscripts are assigned in main factors.

| STRAIN | | Thr | Val | Met | Ile | Leu | Phe | His | Lys | Arg |
| --- | --- | --- | --- | --- | --- | --- | --- | --- | --- | --- |
| NON SEL | | 210±11 | 364±19^b^ | 73±3 | 156±11 | 524±38 | 122±7 | 135±6 | 195±29^b^ | 97±8 |
| SEL | | 212±13 | 305±21^a^ | 77±7 | 140±14 | 494±60 | 134±14 | 145±6 | 109±11^a^ | 116±9 |
| P-value | | ns | P<0.05 | ns | ns | ns | ns | ns | P<0.05 | ns |
|  | |  |  |  |  |  |  |  |  |  |
| TIME | | **Thr** | **Val** | **Met** | **Ile** | **Leu** | **Phe** | **His** | **Lys** | **Arg** |
| 3H | | 193±16 | 336±22^b^ | 63±5 | 139±11^ab^ | 338±35 | 101±10^a^ | 142±12 | 182±28 | 112±11 |
| 6H | | 214±21 | 340±28^ab^ | 66±4 | 141±17^ab^ | 410±34 | 110±5^a^ | 125±7 | 166±39 | 110±11 |
| 12H | | 206±9 | 352±34^b^ | 84±3 | 166±19^ab^ | 624±44 | 147±13^ab^ | 143±6 | 153±51 | 105±8 |
| 18H | | 236±29 | 388±31^b^ | 90±12 | 186±22^b^ | 723±64 | 168±27^b^ | 155±12 | 171±44 | 121±23 |
| 24H | | 205±5 | 234±29^a^ | 73±11 | 99±11^a^ | 477±79 | 117±12^ab^ | 134±9 | 71±7 | 72±7 |
| P-value | | ns | P<0.05 | ns | P<0.05 | P<0.001 | P<0.05 | ns | ns | ns |
|  | |  |  |  |  |  |  |  |  |  |
| STRAIN x TIME | | **Thr** | **Val** | **Met** | **Ile** | **Leu** | **Phe** | **His** | **Lys** | **Arg** |
| NON SEL | 3H | 203±33^a^ | 366±31 | 65±7 | 139±20 | 322±65^ab^ | 91±15 | 136±25 | 222±48 | 110±20 |
| NON SEL | 6H | 234±37^a^ | 395±26 | 72±6 | 173±19 | 478±20^abc^ | 116±7 | 132±11 | 222±66 | 111±14 |
| NON SEL | 12H | 210±16^a^ | 375±64 | 82±3 | 173±36 | 606±59^c^ | 139±11 | 134±8 | 221±90 | 93±12 |
| NON SEL | 18H | 191±17^a^ | 372±49 | 73±4 | 164±24 | 633±37^c^ | 134±17 | 143±12 | 191±73 | 94±27 |
| NON SEL | 24H | 210±4^a^ | 282±18 | 71±18 | 119±4 | 608±54^c^ | 134±17 | 124±15 | 83±3 | 63±13 |
| SEL | 3H | 184±4^a^ | 307±26 | 61±7 | 139±14 | 354±41^a^ | 110±15 | 149±9 | 143±6 | 114±13 |
| SEL | 6H | 194±22^a^ | 284±13 | 60±5 | 108±8 | 341±28^a^ | 103±8 | 117±9 | 110±19 | 109±22 |
| SEL | 12H | 203±12^a^ | 329±34 | 86±6 | 158±22 | 642±77^c^ | 154±25 | 151±6 | 85±5 | 116±3 |
| SEL | 18H | 304±10^b^ | 412±39 | 116±17 | 219±35 | 857±80^d^ | 220±41 | 173±20 | 140±37 | 161±19 |
| SEL | 24H | 200±7^a^ | 187±5 | 75±20 | 80±1 | 346±23^ab^ | 101±9 | 145±1 | 60±3 | 81±1 |
| P-value | | P<0.05 | ns | ns | ns | P<0.01 | ns | ns | ns | ns |

**Table S8.** Free essential amino acid (except tryptophan) mean concentrations n=3 ± SEM in blood plasma (nmol/mL) collected from the caudal vein of two strains of rainbow trout during a 24h period after force feeding of corn protein concentrate. When interaction is present no superscripts are assigned in main factors.

| STRAIN | | Thr | Val | Met | Ile | Leu | Phe | His | Lys | Arg |
| --- | --- | --- | --- | --- | --- | --- | --- | --- | --- | --- |
| NON SEL | | 202±9 | 362±16^b^ | 73±5 | 149±9^b^ | 518±47 | 115±8 | 137±6 | 173±25^b^ | 80±6 |
| SEL | | 182±11 | 292±14^a^ | 78±7 | 119±7^a^ | 453±49 | 116±8 | 139±7 | 80±8^a^ | 81±5 |
| P-value | | ns | P<0.01 | ns | P<0.05 | ns | ns | ns | P<0.01 | ns |
|  | |  |  |  |  |  |  |  |  |  |
| TIME | | **Thr** | **Val** | **Met** | **Ile** | **Leu** | **Phe** | **His** | **Lys** | **Arg** |
| 3H | | 162±19 | 318±20 | 54±6^a^ | 117±7 | 267±19^a^ | 79±3^a^ | 128±16 | 146±24 | 81±5 |
| 6H | | 197±13 | 341±20 | 64±3^ab^ | 132±12 | 371±30^a^ | 99±5^a^ | 128±8 | 150±32 | 90±9 |
| 12H | | 207±16 | 349±31 | 85±6^b^ | 152±17 | 576±27^b^ | 132±7^b^ | 155±8 | 154±51 | 86±12 |
| 18H | | 206±16 | 339±21 | 82±5^b^ | 147±9 | 612±26^b^ | 141±9^b^ | 135±5 | 121±39 | 82±10 |
| 24H | | 196±13 | 288±38 | 91±13^b^ | 122±20 | 601±103^b^ | 127±14^b^ | 144±8 | 62±6 | 64±6 |
| P-value | | ns | ns | P<0.05 | ns | P<0.001 | P<0.001 | ns | ns | ns |
|  | |  |  |  |  |  |  |  |  |  |
| STRAIN x TIME | | **Thr** | **Val** | **Met** | **Ile** | **Leu** | **Phe** | **His** | **Lys** | **Arg** |
| NON SEL | 3H | 181±22 | 352±27 | 58±10 | 122±10 | 273±40 | 76±6 | 126±21 | 190±22 | 86±5 |
| NON SEL | 6H | 208±21 | 383±14 | 64±6 | 156±9 | 419±43 | 100±6 | 122±4 | 195±52 | 82±4 |
| NON SEL | 12H | 228±27 | 390±49 | 89±7 | 175±28 | 603±21 | 136±9 | 159±17 | 228±85 | 94±20 |
| NON SEL | 18H | 190±2 | 372±31 | 73±2 | 160±12 | 619±21 | 127±14 | 143±4 | 184±60 | 84±18 |
| NON SEL | 24H | 204±25 | 311±47 | 80±17 | 130±25 | 675±124 | 135±20 | 132±13 | 69±10 | 54±7 |
| SEL | 3H | 142±30 | 284±13 | 49±5 | 111±9 | 261±13 | 82±1 | 131±29 | 101±18 | 75±8 |
| SEL | 6H | 186±16 | 299±10 | 64±2 | 107±5 | 323±16 | 99±10 | 134±17 | 105±15 | 99±17 |
| SEL | 12H | 186±12 | 307±24 | 80±12 | 130±12 | 548±50 | 127±11 | 150±3 | 80±23 | 79±17 |
| SEL | 18H | 255±1 | 306±17 | 92±6 | 134±12 | 604±54 | 154±8 | 127±6 | 59±4 | 79±11 |
| SEL | 24H | 188±11 | 264±68 | 102±22 | 115±35 | 527±179 | 119±21 | 155±3 | 55±3 | 73±6 |
| P-value | | ns | ns | ns | ns | ns | ns | ns | ns | ns |

**Table S9.** Free essential amino acid (except tryptophan) mean concentrations n=3 ± SEM in blood plasma (nmol/mL) collected from the hepatic portal vein of two strains of rainbow trout during a 24h period after force feeding of wheat gluten meal. When interaction is present no superscripts are assigned in main factors.

| STRAIN | | Thr | Val | Met | Ile | Leu | Phe | His | Lys | Arg |
| --- | --- | --- | --- | --- | --- | --- | --- | --- | --- | --- |
| NON SEL | | 284±30 | 621±50 | 93±14 | 323±40 | 606±63 | 183±20 | 207±37^a^ | 156±26 | 197±43 |
| SEL | | 380±52 | 682±70 | 106±15 | 369±49 | 680±84 | 222±30 | 246±29^b^ | 154±23 | 214±41 |
| P-value | | P<0.01 | ns | ns | ns | ns | ns | P<0.01 | ns | ns |
|  | |  |  |  |  |  |  |  |  |  |
| TIME | | **Thr** | **Val** | **Met** | **Ile** | **Leu** | **Phe** | **His** | **Lys** | **Arg** |
| 3H | | 368±26 | 622±32 | 118±11^b^ | 344±32 | 622±52 | 230±22^bc^ | 260±28^bc^ | 259±15 | 382±38^c^ |
| 6H | | 421±40 | 822±66 | 154±13^b^ | 503±50 | 866±80 | 294±33^c^ | 364±55^d^ | 206±18 | 312±36^c^ |
| 12H | | 502±140 | 869±180 | 131±28^b^ | 472±119 | 896±211 | 247±46^bc^ | 249±48^cd^ | 144±45 | 190±44^b^ |
| 18H | | 223±31 | 499±39 | 50±4^a^ | 222±25 | 442±39 | 111±11^a^ | 122±12^ab^ | 84±11 | 60±6^a^ |
| 24H | | 192±28 | 499±57 | 49±11^a^ | 215±28 | 446±60 | 138±35^ab^ | 129±5^a^ | 67±9 | 56±1^a^ |
| P-value | | P<0.001 | ns | P<0.001 | P<0.001 | P<0.001 | P<0.05 | P<0.001 | P<0.001 | P<0.001 |
|  | |  |  |  |  |  |  |  |  |  |
| STRAIN x TIME | | **Thr** | **Val** | **Met** | **Ile** | **Leu** | **Phe** | **His** | **Lys** | **Arg** |
| NON SEL | 3H | 333±30^ab^ | 597±53^a^ | 105±14 | 308±28^ab^ | 572±59^a^ | 216±20 | 201±15 | 273±19^b^ | 355±56 |
| NON SEL | 6H | 389±40^ab^ | 842±118^a^ | 155±20 | 519±88^bc^ | 899±137^ab^ | 225±36 | 377±107 | 222±22^b^ | 323±66 |
| NON SEL | 12H | 281±102^ab^ | 586±165^a^ | 96±44 | 295±109^ab^ | 584±213^a^ | 205±94 | 191±76 | 73±4^a^ | 129±61 |
| NON SEL | 18H | 234±76^ab^ | 592±1^a^ | 54±1 | 276±31^ab^ | 529±11^a^ | 135±20 | 101±20 | 88±8^a^ | 51±2 |
| NON SEL | 24H | 163±13^a^ | 467±42^a^ | 42±7 | 194±17^a^ | 412±40^a^ | 123±16 | 122±6 | 74±15^a^ | 55±2 |
| SEL | 3H | 403±36^ab^ | 647±42^a^ | 130±17 | 380±57^ab^ | 671±86^a^ | 245±43 | 319±9 | 245±22^b^ | 408±56 |
| SEL | 6H | 453±74^b^ | 802±87^a^ | 152±22 | 486±66^b^ | 834±112^ab^ | 340±24 | 351±56 | 189±28^b^ | 301±45 |
| SEL | 12H | 723±96^c^ | 1152±84^b^ | 167±11 | 650±102^c^ | 1208±166^b^ | 289±15 | 307±33 | 216±47^b^ | 252±12 |
| SEL | 18H | 216±34^ab^ | 437±18^a^ | 48±6 | 186±12^a^ | 385±30^a^ | 104±11 | 135±12 | 81±18^a^ | 66±8 |
| SEL | 24H | 220±53^ab^ | 531±117^a^ | 56±23 | 236±57^ab^ | 479±125^a^ | 152±75 | 135±8 | 61±13^a^ | 57±1 |
| P-value | | P<0.05 | P<0.05 | ns | P<0.05 | P<0.05 | ns | ns | P<0.05 | ns |

**Table S10.** Free essential amino acid (except tryptophan) mean concentrations n=3 ± SEM in blood plasma (nmol/mL) collected from the caudal vein of two strains of rainbow trout during a 24h period after force feeding of wheat gluten meal. When interaction is present no superscripts are assigned in main factors.

| STRAIN | | Thr | Val | Met | Ile | Leu | Phe | His | Lys | Arg |
| --- | --- | --- | --- | --- | --- | --- | --- | --- | --- | --- |
| NON SEL | | 317±45 | 637±52 | 98±13 | 339±39 | 606±63 | 164±18 | 209±37 | 129±22 | 152±28 |
| SEL | | 324±48 | 600±62 | 95±12 | 332±42 | 597±72 | 174±18 | 208±24 | 119±22 | 170±34 |
| P-value | | ns | ns | ns | ns | ns | ns | ns | ns | ns |
|  | |  |  |  |  |  |  |  |  |  |
| TIME | | **Thr** | **Val** | **Met** | **Ile** | **Leu** | **Phe** | **His** | **Lys** | **Arg** |
| 3H | | 362±36^b^ | 549±24 | 109±5^b^ | 279±24^a^ | 493±36^a^ | 183±9^ab^ | 251±34^b^ | 214±6^b^ | 304±28^c^ |
| 6H | | 328±37^b^ | 646±63 | 116±11^b^ | 367±44^a^ | 632±71^a^ | 194±9^ab^ | 295±56^b^ | 145±22^b^ | 219±25^b^ |
| 12H | | 619±63^c^ | 997±73 | 168±8^c^ | 583±56^b^ | 1038±90^b^ | 246±30^b^ | 302±26^b^ | 187±48^b^ | 227±15^b^ |
| 18H | | 202±19^a^ | 539±54 | 67±12^a^ | 273±45^a^ | 498±67^a^ | 120±11^a^ | 116±7^a^ | 61±9^a^ | 59±12^a^ |
| 24H | | 174±25^a^ | 473±53 | 50±11^a^ | 250±41^a^ | 473±71^a^ | 128±32^a^ | 116±4^a^ | 47±5^a^ | 43±1^a^ |
| P-value | | P<0.001 | P<0.001 | P<0.001 | P<0.05 | P<0.05 | P<0.05 | P<0.001 | P<0.001 | P<0.001 |
|  | |  |  |  |  |  |  |  |  |  |
| STRAIN x TIME | | **Thr** | **Val** | **Met** | **Ile** | **Leu** | **Phe** | **His** | **Lys** | **Arg** |
| NON SEL | 3H | 310±7 | 523±28^ab^ | 101±8 | 231±13 | 423±27 | 162±4 | 178±1 | 209±1 | 237±5 |
| NON SEL | 6H | 356±63 | 751±82^bc^ | 134±8 | 438±54 | 744±88 | 183±3 | 359±104 | 181±21 | 240±40 |
| NON SEL | 12H | 564±134 | 892±95^c^ | 157±8 | 515±68 | 925±91 | 250±66 | 312±16 | 176±98 | 224±24 |
| NON SEL | 18H | 228±29 | 638±67^ab^ | 79±22 | 345±69 | 605±98 | 140±20 | 109±15 | 71±16 | 68±26 |
| NON SEL | 24H | 152±8 | 430±34^a^ | 42±7 | 190±16 | 378±32 | 113±13 | 110±6 | 51±8 | 42±1 |
| SEL | 3H | 396±54 | 566±36^ab^ | 114±4 | 311±23 | 540±35 | 197±5 | 300±31 | 218±11 | 349±12 |
| SEL | 6H | 301±46 | 540±47^ab^ | 98±14 | 295±43 | 519±72 | 201±14 | 232±31 | 109±26 | 198±34 |
| SEL | 12H | 674±12 | 1102±29^d^ | 178±10 | 651±72 | 1152±118 | 242±32 | 291±60 | 199±64 | 229±28 |
| SEL | 18H | 176±15 | 441±15^a^ | 55±5 | 201±18 | 391±35 | 107±6 | 124±3 | 52±7 | 51±4 |
| SEL | 24H | 189±43 | 516±104^ab^ | 58±22 | 310±66 | 567±123 | 143±70 | 122±3 | 44±7 | 43±2 |
| P-value | | ns | P<0.05 | ns | ns | ns | ns | ns | ns | ns |

**Table S11.** Free essential amino acid (except tryptophan) mean concentrations n=3 ±SEM in blood plasma (nmol/mL) collected from the hepatic portal vein of two strains of rainbow trout during a 24h period after force feeding of protein blend with and without AA supplementation (Thr, Met and Lys). When interaction is present no superscripts are assigned in main factors.

| **DIET** | **Thr** | **Val** | **Met** | **Ile** | **Leu** | **Phe** | **His** | **Lys** | **Arg** |
| --- | --- | --- | --- | --- | --- | --- | --- | --- | --- |
| MINUS | 176±11 | 296±18 | 30±2 ^a^ | 132±10 | 276±20 | 101±5 | 85±4 | 138±19 | 99±11^a^ |
| PLUS | 223±16 | 328±16 | 98±6 ^b^ | 151±10 | 323±16 | 112±6 | 105±7 | 241±25 | 141±16^b^ |
| P-value | P<0.001 | ns | P<0.001 | ns | P<0.05 | P<0.05 | P<0.01 | P<0.001 | P<0.01 |

| **STRAIN** | **Thr** | **Val** | **Met** | **Ile** | **Leu** | **Phe** | **His** | **Lys** | **Arg** |
| --- | --- | --- | --- | --- | --- | --- | --- | --- | --- |
| NON SEL | 202±13 | 321±15 | 63±8 | 144±9 | 299±17 | 100±5 | 90±5 | 198±21 | 105±12 |
| SEL | 193±15 | 301±20 | 61±9 | 138±11 | 297±21 | 112±6 | 99±7 | 174±27 | 133±16 |
| **P-value** | **ns** | **ns** | **ns** | **ns** | **ns** | **ns** | **ns** | **ns** | **ns** |

| **TIME** | **Thr** | **Val** | **Met** | **Ile** | **Leu** | **Phe** | **His** | **Lys** | **Arg** |
| --- | --- | --- | --- | --- | --- | --- | --- | --- | --- |
| 3H | 225±24 | 339±26 | 64±13 | 145±17 ^b^ | 252±32 | 97±11 | 102±15 | 241±43 | 129±28^b^ |
| 6H | 206±18 | 353±29 | 78±14 | 167±17 ^b^ | 313±28 | 119±7 | 97±5 | 243±36 | 151±22^b^ |
| 12H | 218±21 | 324±18 | 72±16 | 155±13 ^b^ | 352±27 | 122±10 | 103±11 | 180±34 | 147±21^b^ |
| 18H | 204±20 | 310±20 | 59±12 | 146±10 ^b^ | 336±19 | 108±6 | 87±7 | 159±23 | 111±12^ab^ |
| 24H | 137±16 | 231±26 | 38±8 | 94±13 ^a^ | 241±27 | 84±4 | 83±6 | 108±38 | 56±14^a^ |
| **P-value** | **P<0.001** | **P<0.001** | **P<0.001** | **P<0.001** | **P<0.01** | **P<0.001** | **ns** | **P<0.001** | **P<0.01** |

| **DIET X STRAIN** | | **Thr** | **Val** | **Met** | **Ile** | **Leu** | **Phe** | **His** | **Lys** | **Arg** |
| --- | --- | --- | --- | --- | --- | --- | --- | --- | --- | --- |
| MINUS | NON SEL | 179±18 | 315±27 | 31±4 | 140±17 | 289±28 | 96±8 | 80±6 | 150±33 | 87±18 |
| MINUS | SEL | 173±13 | 281±24 | 30±3 | 125±12 | 265±28 | 105±7 | 89±5 | 127±22 | 109±15 |
| PLUS | NON SEL | 224±18 | 327±15 | 93±7 | 148±9 | 308±20 | 104±7 | 99±7 | 242±20 | 122±15 |
| PLUS | SEL | 221±28 | 329±33 | 105±11 | 156±20 | 342±26 | 121±10 | 113±14 | 240±52 | 165±31 |
| **P-value** |  | **ns** | **ns** | **ns** | **ns** | **ns** | **ns** | **ns** | **ns** | **ns** |

| **TIME X DIET** | | **Thr** | **Val** | **Met** | **Ile** | **Leu** | **Phe** | **His** | **Lys** | **Arg** |
| --- | --- | --- | --- | --- | --- | --- | --- | --- | --- | --- |
| MINUS | 3H | 191±14 | 298±20 ^a^ | 37±4 | 118±11 | 200±22 | 79±8 | 83±9 | 174±36 | 92±19 |
| PLUS | 3H | 277±49 | 401±44 ^b^ | 106±17 | 186±29 | 329±56 | 120±20 | 130±33 | 343±71 | 186±57 |
| MINUS | 6H | 214±30 | 390±47 ^b^ | 40±5 | 185±28 | 329±48 | 126±10 | 94±6 | 231±62 | 150±35 |
| PLUS | 6H | 198±23 | 315±32 ^ab^ | 117±12 | 150±18 | 298±35 | 113±9 | 101±7 | 255±44 | 153±32 |
| MINUS | 12H | 190±22 | 309±29 ^ab^ | 32±3 | 143±21 | 337±48 | 111±12 | 91±15 | 121±26 | 119±27 |
| PLUS | 12H | 254±31 | 343±19 ^b^ | 122±8 | 169±15 | 370±18 | 136±14 | 119±14 | 254±50 | 183±26 |
| MINUS | 18H | 172±14 | 298±34 ^ab^ | 25±3 | 143±17 | 324±32 | 107±5 | 78±7 | 114±23 | 93±13 |
| PLUS | 18H | 236±34 | 322±25 ^ab^ | 93±4 | 148±12 | 349±23 | 110±11 | 97±11 | 203±30 | 130±19 |
| MINUS | 24H | 108±11 | 187±18 ^a^ | 16±4 | 74±7 | 204±32 | 82±4 | 80±8 | 42±5 | 42±9 |
| PLUS | 24H | 166±25 | 275±43 ^ab^ | 60±2 | 115±23 | 279±39 | 86±8 | 86±9 | 175±66 | 70±26 |
| **P-value** |  | **ns** | **P<0.05** | **NS** | **ns** | **ns** | **ns** | **ns** | **ns** | **ns** |

| **DIET X STRAIN X TIME** | | | **Thr** | **Val** | **Met** | **Ile** | **Leu** | **Phe** | **His** | **Lys** | **Arg** |
| --- | --- | --- | --- | --- | --- | --- | --- | --- | --- | --- | --- |
| Minus | NON SEL | 3H | 188±6^abc^ | 271±27 | 35±4 | 106±16 | 179±34^a^ | 67±2^a^ | 86±18^a^ | 135±28^abc^ | 69±28 |
| Minus | NON SEL | 6H | 278±18^def^ | 486±27 | 48±2 | 244±16 | 438±1^e^ | 131±23^abc^ | 104±12^a^ | 366±65^d^ | 159±93 |
| Minus | NON SEL | 12H | 142±14^ab^ | 266±7 | 28±4 | 107±4 | 247±17^abc^ | 93±9^abc^ | 61±1^a^ | 82±17^ab^ | 72±1 |
| Minus | NON SEL | 18H | 183±20^abcd^ | 337±8 | 29±3 | 165±6 | 344±9^bcde^ | 113±1^abc^ | 78±12^a^ | 144±23^abc^ | 105±15 |
| Minus | NON SEL | 24H | 97±21^a^ | 223±30 | 12±7 | 87±10 | 264±27^abcd^ | 83±11^abc^ | 71±9^a^ | 34±5^a^ | 29±5 |
| Minus | SEL | 3H | 194±30^abcd^ | 324±24 | 39±7 | 131±15 | 222±26^ab^ | 97±3^abc^ | 80±7^a^ | 212±64^abcd^ | 115±21 |
| Minus | SEL | 6H | 172±27^abcd^ | 326±44 | 35±8 | 146±23 | 256±33^abcd^ | 123±11^abc^ | 86±4^a^ | 141±35^abc^ | 144±33 |
| Minus | SEL | 12H | 221±17^abcd^ | 338±43 | 34±3 | 168±26 | 397±55^de^ | 123±17^abc^ | 111±17^a^ | 147±35^abc^ | 150±34 |
| Minus | SEL | 18H | 155±16^abc^ | 240±75 | 20±2 | 111±30 | 294±91^abcde^ | 97±7^abc^ | 77±4^a^ | 69±16^ab^ | 75±20 |
| Minus | SEL | 24H | 115±14^a^ | 163±9 | 18±6 | 66±6 | 164±35^a^ | 81±4^ab^ | 85±11^a^ | 47±7^a^ | 51±12 |
| PLUS | NON SEL | 3H | 201±23^abcd^ | 358±16 | 80±4 | 155±6 | 263±12^abcd^ | 93±9^abc^ | 82±7^a^ | 269±27^bcd^ | 113±23 |
| PLUS | NON SEL | 6H | 163±10^abc^ | 266±7 | 105±9 | 120±1 | 244±4^abc^ | 100±6^abc^ | 92±8^a^ | 203±33^abcd^ | 104±8 |
| PLUS | NON SEL | 12H | 299±16^ef^ | 353±1 | 134±6 | 174±10 | 385±19^cde^ | 150±17^c^ | 131±25^a^ | 255±9^bcd^ | 197±4 |
| PLUS | NON SEL | 18H | 274±46^bcde^ | 357±5 | 95±6 | 164±3 | 356±34^bcde^ | 94±11^abc^ | 99±18^a^ | 248±14^bcd^ | 130±30 |
| PLUS | NON SEL | 24H | 200±25^abcd^ | 320±55 | 60±2 | 137±33 | 303±60^abcde^ | 95±10^abc^ | 96±13^a^ | 249±86^bcd^ | 88±42 |
| PLUS | SEL | 3H | 353±49^f^ | 443±89 | 132±19 | 217±57 | 396±100^de^ | 146±29^bc^ | 178±43^b^ | 416±136^d^ | 258±92 |
| PLUS | SEL | 6H | 251±24^bcd^ | 389±32 | 135±27 | 193±14 | 378±33^cde^ | 132±14^abc^ | 114±1^a^ | 332±77^bcd^ | 226±29 |
| PLUS | SEL | 12H | 209±39^abcd^ | 334±45 | 109±1 | 164±35 | 354±32^bcde^ | 123±23^abc^ | 106±14^a^ | 252±123^bcd^ | 169±61 |
| PLUS | SEL | 18H | 179±10^abcd^ | 270±42 | 91±7 | 124±22 | 338±41^bcde^ | 133±6^abc^ | 95±13^a^ | 136±28^abc^ | 129±30 |
| PLUS | SEL | 24H | 115±4^a^ | 208±41 | 59±5 | 80±17 | 242±50^abc^ | 73±9^a^ | 72±1^a^ | 63±8^a^ | 44±9 |
| **P-value** |  |  | **P<0.001** | **ns** | **ns** | **ns** | **P<0.01** | **P<0.05** | **P<0.01** | **P<0.05** | **ns** |

**Table S12.** Free essential amino acid (except tryptophan) mean concentrations n=3 ±SEM in blood plasma (nmol/mL) collected from the caudal vein of two strains of rainbow trout during a 24h period after force feeding of protein blend with and without AA supplementation (Thr, Met and Lys). When interaction is present no superscripts are assigned in main factors.

| **DIET** | **Thr** | **Val** | **Met** | **Ile** | **Leu** | **Phe** | **His** | **Lys** | **Arg** |
| --- | --- | --- | --- | --- | --- | --- | --- | --- | --- |
| **MINUS** | 164±8 | 353±18 | 37±3 | 144±9 | 322±19 | 105±4 | 104±5 | 112±8 | 105±6**^a^** |
| **PLUS** | 216±14 | 354±17 | 118±4 | 149±8 | 337±14 | 114±3 | 129±6 | 233±22 | 148±10**^b^** |
| P-value | P<0.001 | ns | P<0.001 | ns | ns | ns | P<0.01 | P<0.001 | P<0.001 |
| **STRAIN** | **Thr** | **Val** | **Met** | **Ile** | **Leu** | **Phe** | **His** | **Lys** | **Arg** |
| **NON SEL** | 212±12 | 389±16 | 79±9 | 164±9 | 352±16 | 111±4 | 119±6 | 210±21 | 132±9 |
| **SEL** | 171±11 | 324±16 | 77±9 | 131±7 | 310±16 | 109±4 | 114±6 | 143±19 | 121±10 |
| P-value | P<0.001 | P<0.01 | ns | P<0.05 | ns | ns | ns | P<0.001 | ns |

| **TIME** | **Thr** | **Val** | **Met** | **Ile** | **Leu** | **Phe** | **His** | **Lys** | **Arg** |
| --- | --- | --- | --- | --- | --- | --- | --- | --- | --- |
| **3H** | 230±20 | 410±19 | 84±11 | 159±9 | 292±21 | 100±7 | 130±13 | 233±35 | 145±20 ^b^ |
| **6H** | 180±13 | 365±30 | 90±15 | 152±16 | 308±29 | 114±4 | 115±6 | 183±26 | 135±12 ^b^ |
| **12H** | 170±15 | 311±14 | 77±14 | 132±8 | 327±20 | 115±5 | 115±8 | 135±16 | 117±7 **^ab^** |
| **18H** | 191±24 | 342±24 | 73±14 | 147±12 | 365±20 | 111±5 | 111±9 | 146±27 | 124±14 **^ab^** |
| **24H** | 174±20 | 332±40 | 61±13 | 140±19 | 360±37 | 106±8 | 111±11 | 169±51 | 106±18 **^a^** |
| P-value | P<0.001 | P<0.01 | P<0.01 | ns | ns | ns | ns | P<0.001 | P<0.01 |

| **DIET x STRAIN** | | **Thr** | **Val** | **Met** | **Ile** | **Leu** | **Phe** | **His** | **Lys** | **Arg** |
| --- | --- | --- | --- | --- | --- | --- | --- | --- | --- | --- |
| MINUS | NON SEL | 175±10 | 386±24 | 40±4 | 163±14 | 349±25 | 105±6 | 102±8 | 140±11 | 109±9 |
| MINUS | SEL | 154±11 | 323±24 | 34±4 | 126±10 | 296±28 | 105±6 | 106±7 | 88±7 | 100±8 |
| PLUS | NON SEL | 252±17 | 391±23 | 121±6 | 166±12 | 355±22 | 116±4 | 137±6 | 281±28 | 157±13 |
| PLUS | SEL | 187±19 | 324±22 | 117±6 | 136±10 | 323±17 | 112±5 | 122±10 | 194±30 | 141±16 |
| P-value |  | ns | ns | ns | ns | ns | ns | ns | ns | ns |
|  |  |  |  |  |  |  |  |  |  |  |
| **STRAIN X TIME** | | **Thr** | **Val** | **Met** | **Ile** | **Leu** | **Phe** | **His** | **Lys** | **Arg** |
| NON SEL | 3H | 225±17 | 397±25 | 77±13 | 158±12 | 285±27 | 96±8 | 128±11 | 236±45 | 125±28 |
| NON SEL | 6H | 193±16 | 396±51 | 88±20 | 169±28 | 342±43 | 113±5 | 122±8 | 193±20 | 130±10 |
| NON SEL | 12H | 177±29 | 306±15 | 83±28 | 123±10 | 308±22 | 120±10 | 113±19 | 132±22 | 116±11 |
| NON SEL | 18H | 237±40 | 402±23 | 82±24 | 178±12 | 395±23 | 108±8 | 108±14 | 213±35 | 149±22 |
| NON SEL | 24H | 221±27 | 430±35 | 58±18 | 186±19 | 429±17 | 125±5 | 126±22 | 272±93 | 135±30 |
| SEL | 3H | 234±35 | 420±30 | 89±18 | 160±15 | 298±34 | 105±11 | 132±24 | 230±56 | 161±28 |
| SEL | 6H | 168±20 | 335±30 | 93±26 | 135±16 | 274±36 | 114±6 | 108±10 | 174±47 | 140±24 |
| SEL | 12H | 166±18 | 315±21 | 73±17 | 137±12 | 340±31 | 112±6 | 116±8 | 136±23 | 118±9 |
| SEL | 18H | 145±14 | 283±26 | 64±17 | 116±12 | 334±30 | 115±8 | 114±11 | 79±10 | 98±12 |
| SEL | 24H | 137±17 | 253±40 | 64±21 | 103±19 | 304±56 | 95±10 | 100±6 | 87±21 | 82±18 |
| P-value |  | P<0.05 | P<0.05 | ns | P<0.05 | ns | ns | ns | P<0.001 | ns |

| **DIET X TIME** | | **Thr** | **Val** | **Met** | **Ile** | **Leu** | **Phe** | **His** | **Lys** | **Arg** |
| --- | --- | --- | --- | --- | --- | --- | --- | --- | --- | --- |
| MINUS | 3H | 196±15 | 379±28 | 53±3 | 139±9 | 248±19 | 85±3 | 116±13 | 140±21 | 102±18 |
| PLUS | 3H | 272±32 | 447±16 | 120±8 | 184±9 | 345±26 | 120±9 | 146±25 | 344±21 | 195±23 |
| MINUS | 6H | 157±21 | 398±55 | 41±6 | 164±32 | 316±56 | 111±6 | 99±8 | 116±20 | 111±15 |
| PLUS | 6H | 204±8 | 333±20 | 139±8 | 140±9 | 300±21 | 117±5 | 131±4 | 238±30 | 159±15 |
| MINUS | 12H | 166±16 | 333±11 | 36±4 | 143±6 | 361±20 | 114±8 | 108±12 | 107±5 | 114±6 |
| PLUS | 12H | 175±27 | 289±21 | 118±9 | 120±14 | 294±30 | 117±7 | 121±12 | 162±26 | 121±13 |
| MINUS | 18H | 157±8 | 345±21 | 29±4 | 147±11 | 369±25 | 113±8 | 102±12 | 105±16 | 108±5 |
| PLUS | 18H | 224±46 | 340±47 | 117±9 | 146±24 | 360±34 | 110±8 | 119±13 | 187±47 | 139±28 |
| MINUS | 24H | 136±23 | 286±67 | 21±4 | 118±32 | 322±76 | 107±22 | 89±10 | 81±20 | 81±19 |
| PLUS | 24H | 205±26 | 368±48 | 93±7 | 157±23 | 390±32 | 106±7 | 129±12 | 239±81 | 126±27 |
| P-value |  | ns | P<0.05 | ns | P<0.05 | ns | ns | ns | P<0.01 | ns |

| **DIET X STRAIN X TIME** | | | **Thr** | **Val** | **Met** | **Ile** | **Leu** | **Phe** | **His** | **Lys** | **Arg** |
| --- | --- | --- | --- | --- | --- | --- | --- | --- | --- | --- | --- |
| MINUS | NON SEL | 3H | 208±13^abcde^ | 360±19^abc^ | 57±5^c^ | 144±15^abc^ | 253±34^abc^ | 83±3 | 134±20^ab^ | 171±33^abc^ | 96±37 |
| MINUS | NON SEL | 6H | 188±35^abcde^ | 479±74^c^ | 46±10^bc^ | 208±49^c^ | 402±71^de^ | 111±11 | 108±10^ab^ | 154±21^abc^ | 123±18 |
| MINUS | NON SEL | 12H | 138±1^ab^ | 319±26^abc^ | 36±3^abc^ | 131±8^abc^ | 321±32^abcde^ | 111±13 | 84±4^a^ | 106±15^ab^ | 100±3 |
| MINUS | NON SEL | 18H | 154±10^abcd^ | 365±29^abc^ | 30±5^ab^ | 159±15^abc^ | 363±36^abcde^ | 109±16 | 82±6^a^ | 138±11^abc^ | 111±8 |
| MINUS | NON SEL | 24H | 174±7^abcde^ | 387±60^bc^ | 27±4^ab^ | 164±35^abc^ | 422±3^de^ | 135±3 | 92±24^ab^ | 114±19^ab^ | 112±10 |
| MINUS | SEL | 3H | 183±27^abcde^ | 397±57^bc^ | 49±4^bc^ | 134±12^abc^ | 244±24^abc^ | 86±5 | 86±3^a^ | 109±9^ab^ | 109±12 |
| MINUS | SEL | 6H | 125±9^ab^ | 317±56^abc^ | 37±7^abc^ | 120±29^abc^ | 229±56^ab^ | 111±8 | 90±12^ab^ | 91±20^ab^ | 98±24 |
| MINUS | SEL | 12H | 185±20^abcde^ | 342±9^abc^ | 36±7^abc^ | 151±4^abc^ | 388±9^cde^ | 116±12 | 123±13^ab^ | 108±2^ab^ | 124±4 |
| MINUS | SEL | 18H | 161±13^abcde^ | 325±31^abc^ | 28±7^ab^ | 135±14^abc^ | 374±42^abcd^ | 116±10 | 122±15^ab^ | 73±7^ab^ | 106±6 |
| MINUS | SEL | 24H | 98±18^a^ | 185±58^a^ | 16±4^a^ | 71±27^a^ | 222±121^a^ | 93±29 | 86±4^a^ | 49±2^a^ | 49±15 |
| PLUS | NON SEL | 3H | 251±35^cde^ | 453±8^c^ | 107±6^de^ | 181±2^bc^ | 333±5^abcde^ | 114±6 | 118±3^ab^ | 333±40^ef^ | 168±28 |
| PLUS | NON SEL | 6H | 198±9^abcde^ | 313±20^abc^ | 130±9^ef^ | 130±8^abc^ | 282±25^abcd^ | 116±5 | 135±5^ab^ | 219±18^bcd^ | 136±11 |
| PLUS | NON SEL | 12H | 216±43^bcde^ | 293±19^abc^ | 130±18^ef^ | 116±21^abc^ | 296±39^abcde^ | 129±16 | 141±23^ab^ | 159±36^abc^ | 132±15 |
| PLUS | NON SEL | 18H | 320±30^f^ | 439±18^bc^ | 134±8^f^ | 196±12^c^ | 428±18^de^ | 107±8 | 133±18^ab^ | 289±15^de^ | 187±31 |
| PLUS | NON SEL | 24H | 267±6e^f^ | 473±5^c^ | 89±3^d^ | 208±4^c^ | 437±40^e^ | 120±3 | 159±5^b^ | 431±43^f^ | 157±64 |
| PLUS | SEL | 3H | 285±53^f^ | 443±28^bc^ | 128±9^ef^ | 186±17^c^ | 353±46^abcde^ | 123±16 | 201±26^c^ | 351±30^f^ | 214±32 |
| PLUS | SEL | 6H | 210±13^bcde^ | 354±34^abc^ | 148±12^f^ | 149±16^abc^ | 318±34^abcde^ | 118±9 | 126±5^ab^ | 257±61^cde^ | 182±22 |
| PLUS | SEL | 12H | 147±28^abcd^ | 287±38^abc^ | 110±8^de^ | 123±22^abc^ | 293±49^abcde^ | 108±2 | 108±10^ab^ | 164±43^abc^ | 113±20 |
| PLUS | SEL | 18H | 129±23^ab^ | 240±23^ab^ | 100±6^d^ | 96±12^b^ | 293±33^abcde^ | 113±15 | 105±17^ab^ | 86±20^ab^ | 91±25 |
| PLUS | SEL | 24H | 163±3^abcde^ | 299±40^abc^ | 96±12^d^ | 124±20^abc^ | 359±42^abcde^ | 96±6 | 109±2^ab^ | 112±27^ab^ | 104±20 |
| P-value |  |  | P<0.01 | P<0.01 | P<0.05 | P<0.01 | P<0.05 | ns | P<0.01 | P<0.001 | ns |
